# Putrescine supplementation shifts macrophage L-arginine metabolism related-genes reducing *Leishmania amazonensis* infection

**DOI:** 10.1371/journal.pone.0283696

**Published:** 2023-03-31

**Authors:** Jonathan Miguel Zanatta, Stephanie Maia Acuña, Yan de Souza Angelo, Camilla de Almeida Bento, Jean Pierre Schatzman Peron, Beatriz Simonsen Stolf, Sandra Marcia Muxel

**Affiliations:** 1 Departamento de Fisiologia, Instituto de Biociências, Universidade de São Paulo, São Paulo, Brazil; 2 Departamento de Imunologia, Instituto de Ciências Biomédicas, Universidade de São Paulo, São Paulo, Brazil; 3 Departamento de Parasitologia, Instituto de Ciências Biomédicas, Universidade de São Paulo, São Paulo, Brazil; Instituto Oswaldo Cruz, BRAZIL

## Abstract

*Leishmania* is a protozoan that causes leishmaniasis, a neglected tropical disease with clinical manifestations classified as cutaneous, mucocutaneous, and visceral leishmaniasis. In the infection context, the parasite can modulate macrophage gene expression affecting the microbicidal activity and immune response. The metabolism of L-arginine into polyamines putrescine, spermidine, and spermine reduces nitric oxide (NO) production, favoring *Leishmania* survival. Here, we investigate the effect of supplementation with L-arginine and polyamines in infection of murine BALB/c macrophages by *L*. *amazonensis* and in the transcriptional regulation of genes involved in arginine metabolism and proinflammatory response. We showed a reduction in the percentage of infected macrophages upon putrescine supplementation compared to L-arginine, spermidine, and spermine supplementation. Unexpectedly, deprivation of L-arginine increased nitric oxide synthase (*Nos*2) gene expression without changes in NO production. Putrescine supplementation increased transcript levels of polyamine metabolism-related genes *Arg*2, ornithine decarboxylase *(Odc1)*, Spermidine synthase (*SpdS)*, and Spermine synthase (*SpmS*), but reduced *Arg1* in *L*. *amazonensis* infected macrophages, while spermidine and spermine promoted opposite effects. Putrescine increased *Nos2* expression without leading to NO production, while L-arginine plus spermine led to NO production in uninfected macrophages, suggesting that polyamines can induce NO production. Besides, L-arginine supplementation reduced *Il-1b* during infection, and L-arginine or L-arginine plus putrescine increased *Mcp1* at 24h of infection, suggesting that polyamines availability can interfere with cytokine/chemokine production. Our data showed that putrescine shifts L-arginine-metabolism related-genes on BALB/c macrophages and affects infection by *L*. *amazonensis*.

## 1. Introduction

Macrophage response and polarization may determine the inflammatory process and the outcome of infection by different pathogens. Parasitic infections can modify macrophage metabolism and the activity of enzymes such as those from L-arginine and polyamines pathways. Polyamines play essential roles in macrophage activation by regulating amino acid and protein synthesis, oxidative DNA damage, histone modifications, chromatin structure, and tricarboxylic acid (TCA) cycle [1–3]. The polyamines putrescine, spermidine, and spermine are aliphatic polycations with low molecular weight [4]. The cytosolic enzyme arginase 1 (ARG1) and the mitochondrial isoform arginase 2 (ARG2) convert L-arginine in ornithine and urea [5]. Ornithine is then converted by ornithine decarboxylase (ODC) into putrescine, which is subsequently transformed in spermidine by spermidine synthase (SPDS), and in spermine by spermine synthase (SPMS) [6]. Macrophage ARG1 uses L-arginine to produce ornithine and, subsequently, polyamines, driving cell proliferation, collagen synthesis, tissue repair, and wound healing [7, 8].

ARG1 can impair nitric oxide (NO) production by competing for L-arginine. Nitric oxide synthase 2 (NOS2) consumes L-arginine and oxygen to generate citrulline and NO production in response to interferon-gamma (IFN-ɣ) and lipopolysaccharide (LPS), resulting in high microbicidal activity in macrophages [9, 10]. The antagonism between ARG1 and NOS2 affects the microbicidal function of macrophages but also regulates the inflammation mediated by NOS2/NO, the proliferation of T cells, pathogen escape from immune response, and tissue repair and wound healing [11–17].

L-arginine and polyamines are valuable resources for macrophages, internalized via transporters from the amino acid-polyamine-organocation (APC) superfamily, including cationic amino acid transporters such as CAT1/SLC7A1 and CAT2/SLCA2, and members of the solute carrier transporters (SLC’s) family [18]. Interestingly, polyamines are uptaken and exported via heterodimeric transporter SLC3A2/SLC7A5 at different levels in M1/M2 polarized macrophages compared to resting cells [19–22]. The SLC25A15, SLC1A5, SLC7A5, and SLC3A2 transporters regulate the uptake of L-arginine, L-glutamine, L-ornithine and polyamines in macrophages [19, 20, 22, 23]. Thus the amounts of such transporters can modulate L-arginine metabolism and the outcome of infection [24, 25].

Leishmaniasis is a parasitic disease caused by *Leishmania*, endemic in the Americas, Africa, Europe, and Asia. It is characterized by a large spectrum of clinical manifestations grouped into cutaneous, mucocutaneous, or visceral forms [26–28]. The absence of effective treatment or vaccines reflects low public health policy commitment and is responsible for the high death rate of visceral leishmaniasis [29].

*Leishmania amazonensis* is one of Brazil’s causative agents of cutaneous leishmaniasis [27, 28]. *Leishmania* live as promastigotes in the sand fly invertebrate host from the *Phlebotomus* or *Lutzomyia* and as amastigotes in mammals such as rodents and humans [30–33]. Promastigotes are transmitted to mammals during the bite of an infected sandfly, and interact and are phagocytosed by resident macrophages in the region of the injury. Once inside the phagolysosome, the parasite differentiates into the amastigote form and multiplies [34].

The balance between macrophage activation and parasite ability to subvert the host proinflammatory response determines the fate of *Leishmania* infection [35, 36]. One well-known mechanism of subversion is by consuming L-arginine and modulating gene expression of proteins related to polyamines synthesis [25, 37, 38]. Indeed, *Leishmania* is auxotrophic for L-arginine, but can regulate its content inside macrophages, interfering in its metabolism [34, 37, 39–41]. Besides, polyamines are essential for host-glutathione and parasite-trypanothione biosynthesis. Thus, these pathways compete for polyamines and L-arginine during infection, interfering in NO production and microbicidal activity [42, 43].

Here we analyzed the influence of L-arginine and polyamines in modulating their metabolism-related genes and *L*. *amazonensis* infection in BALB/c-macrophages. We found a reduced percentage of infected macrophages upon putrescine supplementation compared to arginine, spermidine, or spermine supplementation. Also, putrescine supplementation increased *Arg*2, *Odc1*, *Spd*S, *Spm*S, and *Nos*2 transcripts without leading to NO production. On the other hand, L-arginine plus spermine increased NO production in uninfected macrophages. Also, putrescine supplementation increased the levels of *Il1b* and *Mcp1*. Our data support that polyamines affect macrophage activation and control of *Leishmania* infection, not necessarily associated with NO production.

## 2. Methods

### 2.1. Ethics statement

The experimental protocol for the animal experiments was approved by the Comissão de Ética no Uso de Animais (CEUA) from the Instituto de Biociências of the Universidade de São Paulo (approval number CEUA-IB: IB-USP 314/2018). For all experiments requiring the use of bone marrow cell, mice were euthanized using isoflurane chamber, ensuring that mice were completely dead before removal from the chamber, in strict accordance with the recommendations in the guide and policies for the care and use of laboratory animals of São Paulo State (Lei Estadual 11.977, de 25 August 2005) and the Brazilian government (Lei Federal 11.794, de 8 October 2008).

### 2.2. Murine macrophages differentiation

Bone marrow cells were isolated from femurs and tibiae of BALB/c female mice aged 6 to 8 weeks, supplied by the Centro de Biotério da Faculdade de Medicina da Universidade de São Paulo and maintained at the Instituto de Biociências da USP. Cells were incubated with 10% supernatant from the conditioned culture of L929 cells in RPMI 1640 medium (LGC, São Paulo, Brazil), supplemented with 10% inactivated fetal bovine serum, 50 U penicillin, 50 μg / mL streptomycin (Gibco™, USA) for 7 days at 34° C and 5% CO_2_.

### 2.3. Macrophage infection and polyamine treatment

Promastigotes of *L*. *amazonensis* (MHOM / BR / 1973 / M2269) were maintained in M199 medium (Gibco) supplemented with 10% heat-inactivated fetal bovine serum (Invitrogen), 5 mg/L hemine, 100 μM adenine, 100 U penicillin, 100 μg/mL streptomycin, 40 mM Hepes-NaOH, and 12 mM NaHCO3, at pH 6.85, at 25°C for a week-long culture at low passage numbers (up to 5).

For RNA analysis, 5 x 10^6^ macrophages/well were plated in 6-well plates (SPL Lifescience, Korea); for flow cytometry analysis, 1 x 10^6^ macrophages/well were plated in 24-well plates, and for infectivity, 2 x 10^5^ macrophages/well were plated in 8-well chamber slides (Sigma, USA). Macrophages were maintained for 18 h at 34° C, 5% CO_2_. Then, macrophages and stationary-phase promastigote forms of *L*. *amazonensis* (La) were washed twice with 1X PBS and co-cultured at a ratio of 5 parasites per macrophage (MOI 5:1) in RPMI 1640 medium without L-arginine (R1780, Sigma-Aldrich, USA) supplemented with 2% of heat-inactivated fetal bovine serum (FBS) (Invitrogen) with or without L-arginine, putrescine, spermidine, and spermine, as follows: deprived of L-arginine (arg-); supplemented with L-arginine (400 μM, arg+); supplemented with putrescine (100 μM, put+); supplemented with L-arginine plus putrescine (arg+/put+); supplemented with spermidine (100 μM, spd+); supplemented with L-arginine plus spermidine (arg+/spd+); supplemented with spermine (100 μM, spm+); supplemented with L-arginine plus spermine (arg+/spm+). These conditions were employed for uninfected (MO) and infected macrophages (MO-La).

After 4 hours of infection, the culture was washed twice with 1X PBS to remove non-phagocyted parasites. Then, cells were maintained with complete RPMI 1640 medium (LGC, São Paulo, Brazil) supplemented with 10% inactivated FBS, 50 U penicillin, 50 μg / mL streptomycin at 34° C and 5% CO_2_. For the infection analysis, the culture was maintained for 24 and 48 hours, cells in glass slides were fixed with acetone: methanol (1: 1, v: v), stained with Panoptic (Laborclin, Parana, Brazil), and infectivity was analyzed by optical microscopy. The percentage of infected macrophages and the number of amastigotes per infected macrophage were calculated by randomly counting at least 500 macrophages per slide.

### 2.4. RNA extraction and reverse transcription

Macrophages were washed 2-times with 1x PBS. The supernatant was discarded, macrophages were resuspended in 750μL of Trizol^TM^ reagent (Invitrogen), and RNA extraction was performed following the manufacturer´s instructions. The RNA was resuspended in 20μL of RNAse-free water and quantified by spectrometry (NanoDrop, Thermo Fisher Scientific). cDNA synthesis was performed using the RevertAID Reverse Transcriptase kit (ThermoScientific), following the manufacturer’s instructions. Briefly, the reaction was prepared with 2 μg of total RNA, 2 μL of random primer oligos (1.5 μg/ μL, ThermoScientific), 2 μL of dNTP (10mM, ThermoScientific), and water q.s.p. 26 μL, and incubated at 72°C for 5 min. Then, 8 μL of 5x Buffer, 2 μL of DTT (0.1M), 2 μL RNAse OUT and 2 μL of reverse transcriptase (200U / μL) were added and the samples were incubated at 37°C for 5 min, 25°C for 10 min, 42°C for 45min and 72°C for 10 min. The negative controls of reverse transcription were prepared with the samples under the same conditions without reverse transcriptase to discard possible contamination by genomic DNA. The obtained cDNAs were diluted 10 times in RNAse-free water for qPCR.

### 2.5. Relative quantification of mRNA by RT-qPCR

The reaction was assembled with 2X SYBR Green PCR Master Mix, 200 nM of oligonucleotides, and 5 μL of template cDNA (10x diluted) in a final volume of 10 μL. The oligonucleotide pairs used are shown in Table 1. The reactions were performed using *StepOne* Real-Time PCR System (*Applied Biosystems*, Thermo Fisher Scientific): the first incubation at 95° C for 10 minutes and 40 cycles of 94° C for the 30s and 60° C for 30s. To evaluate the qPCR efficiency, standard curves containing the target fragment cloned in pGEM T-Easy were used in 10x serial dilution from 10^8^ to 10^2^ molecules, resulting in an efficiency of 95–105%. The fold-change was calculated by the *Delta*-*Delta Ct* (ΔΔCt) method, normalizing the gene expression by housekeeping gene β-2-microglobulin and calculating the relative gene expression with the mean values from group macrophage non-infected and supplemented with arginine for 4 h (arg+ 4h). The fold-change was presented in log2 of mean values and SEM.

**Table 1:** List of oligonucleotide pairs.

*Target gene*	NCBI Reference Sequence	Primer	Sequence
Cationic amino acid transporter 1 (*Cat1*)	NM_001301424.1	Forward	cgtaatcgccactgtgacct
		Reverse	ggctggtaccgtaagaccaa
Cationic amino acid transporter 2 (*Cat2*)	XM_006509252.4	Forward	tccaaaacgaagacaccagt
		Reverse	gccatgagggtgccaataga
Solute Carrier Family 3 Member 2 (*Slc3a2*)	XM_017318078.3	Forward	gccactgagaatgcaaagacc
		Reverse	ttcacgacgtgatgggatgt
Solute Carrier Family 7 Member 5 (*Slc7a5*)	NM_011404.3	Forward	aagggcagggattcatggtg
		Reverse	gtaggggtgtctttcagggc
Solute Carrier Family 1 Member 5 (*Slc1a5*)	NM_009201.2	Forward	cacttcctgtgaccttccca
		Reverse	actctagggccatggtcaatac
Solute Carrier Family 25 Member 15 (*Slc25a15*)	NM_181325.4	Forward	gcgaccttaaaaattgcccg
		Reverse	ctggtttctgtggaaggcga
Arginase 1 (*Arg1*)	NM_007482.3	Forward	agcactgaggaaagctggtc
		Reverse	cagaccgtgggttcttcaca
Arginase 2 (*Arg2*)	NM_009705.3	Forward	tctcctccacgggcaaattc
		Reverse	cactcctagcttcttctgccc
Ornithine decarboxilase (*Odc1*)	NM_013614.3	Forward	ctgccagtaacggagtccag
		Reverse	tcagtggcaatccgtagaacc-
Spermidine Synthase (*SpdS*)	NM_009272.4	Forward	tggtggactacgcctactgt
		Reverse	tggtgcggtttttgcta
Spermine Synthase (*SpmS*)	NM_009214.4	Forward	acactatggcagcagcaagac
		Reverse	tgtgcactgactctgtcatcc
Nitric Oxide Synthase (*Nos2*)	NM_010927.4	Forward	agagccacagtcctctttgc
		Reverse	gctcctcttccaaggtgctt
Interleukin-1-β (*Il-1b*)	NM_008361.4	Forward	ccaagcttccttgtgcaagtg
		Reverse	ctgtcaaaaggtggcatttcac
Tumor Necrosis Factor alpha (*Tnfα*)	NM_013693.3	Forward	ccaccacgctcttctgtcta
		Reverse	agggtctgggccatagaact
Monocyte Chemoattractant Protein-1 (*Mcp1*)	NM_011333.3	Forward	tgatcccaatgagtaggctgg
		Reverse	gcacagacctctctcttgagc
β-2-microglobulin (*B2M*)	AH001856.2	Forward	cactgaattcacccccactga
		Reverse	acagatggagcgtccagaaag

### 2.6. NO quantification assay

5 x 10^6^ macrophages/well were plated in 6-well plates (SPL Lifescience, Korea) and infected in the conditions described above. Macrophages were detached by incubation with 200 uL of 1mM EDTA in 1X PBS for 10 min at 34°C, then adding RPMI plus 10% FBS and cell scraping on ice. The cells were transferred to a new 96-well V-bottom plate and washed by centrifugation with cold 1X PBS (500 x g, 10 min, 4°C) and incubated with 50 μL of 5 μM DAF-FM (4-amino-5methylamino-2’,7’-dichlorofluorescein diacetate, Life Technologies, Eugene, OR, USA) diluted in 1X PBS for 30 min at 34°C. Cells were then washed with 1X PBS, centrifuged (500 x g, 10 min, 4°C) and resuspended in 300 μL of cold 1X PBS. Fluorescence acquisition was performed using BD Accuri C6 cytometer (BD, Franklin Lakes, NJ, USA), and the collected data were analyzed by gating cells based on the characteristics of forward scatter (FSC) and side scatters (SSC) for 20,000 events, and gating DAF-FM+ cells in FL1 detector. We used unlabeled cells and LPS (100 ng/mL) plus IFN-γ (50ng/mL) as controls.

### 2.7. Flow cytometry for NOS2, IL1F062 and TNF

3 x 10^6^ macrophages/well were plated in 6-well plates (SPL Lifescience, Korea) and infected in the conditions described above. For the intracellular label of IL1b and TNF, we added 1 uL of Brefeldin A (Biolegend) 4h before collecting the cells at 4 and 24h of infection. Macrophages were detached by incubation with 200 uL of 1mM EDTA in 1X PBS for 10 min at 34°C, then adding RPMI plus 10% FBS and cell scraping on ice. The cells were transferred to a new 96-well V-bottom plate and fixed and permeabilized with BD Cytofix/Cytoperm and BD Permewash (BD Bioscience), following the manufacturer’s instruction. The cells were incubated with 25 μL of FITC-anti-NOS2, PE-anti-TNF, and APC-anti-IL1b pro-form (BD Bioscience, USA) diluted in 1:200 in PBS for 1 h at room temperature. Cells were then washed by centrifugation with 1X PBS (500 x g, 10 min, 4°C) and resuspended in 300 μL of cold 1X PBS. Fluorescence acquisition was performed using BD Accuri C6 cytometer (BD, Franklin Lakes, NJ, USA), and the collected data were analyzed by gating cells based on the characteristics of forward scatter (FSC) and side scatters (SSC) for 20,000 events, and gating NOS+ cells in FL1 detector, TNF+ cells in FL2 detector and IL1B+ cells in FL3 detector. We used unlabeled cells and LPS (100 ng/mL) as controls.

### 2.8. Statistical analysis

The collected data were analyzed in GraphPad Prism software 7. The statistical analyses were performed using One-way ANOVA for mRNA and cytometry analysis and Two-way ANOVA for infectivity, with a 95% confidence interval followed by Sidak’s post-hoc test. Comparisons were based on the groups supplemented with L-arginine uninfected and infected (MO/arg+ and MO-La/arg+, respectively). Comparisons of uninfected and infected groups were performed between similar conditions. Comparisons within a single group (uninfected or infected groups) were based on deprived x supplemented conditions for the same polyamine. For the compiled data table creation, the data was first submitted to a normality test and cleaned from outliers using Grubbs’ test (a = 0.05) before any posterior analysis. For the Principal Component Analysis (PCA) confection, the data was tabled and then submitted to the package missMDA to iterate missing data and add power to the analysis. The complete data was then submitted to ggfortify (0.4.14) for the generation of the actual PCA. The correlation plots were generated by GGally (1.5.0) or ggstatplot (0.9.0), and the proper correlation tests (Spearman or Pearson) were conducted with an established 95% confidence interval. The heatmaps were generated by the pheatmap package (1.0.12), including the so represented data. NA values were omitted and not used, with the exception of the PCA analysis.

## 3. Results

### 3.1. Polyamines affect macrophage infection

Initially, we evaluated the impact of polyamines and arginine supplementation on the infection of BALB/c Bone Marrow-Derived Macrophages (BMDM) by *L*. *amazonensis* as described above. To choose the concentrations used for supplementation, we considered data from our previous works, where we showed that BALB/c-peritoneal macrophages after 24h of infection with *L*. *amazonensis* can uptake a maximum of 600 nM of L-arginine after 15–30 min of incubation with 100 μM of L-arginine [44]. *L*. *amazonensis* promastigotes can uptake a maximum of 500 nM of L-arginine after 120 min of incubation with 50 μM of L-arginine [45], and the rate of L-arginine uptake by the parasite is similar when supplemented with 400 μM of L-arginine [35].

**Fig 1:**
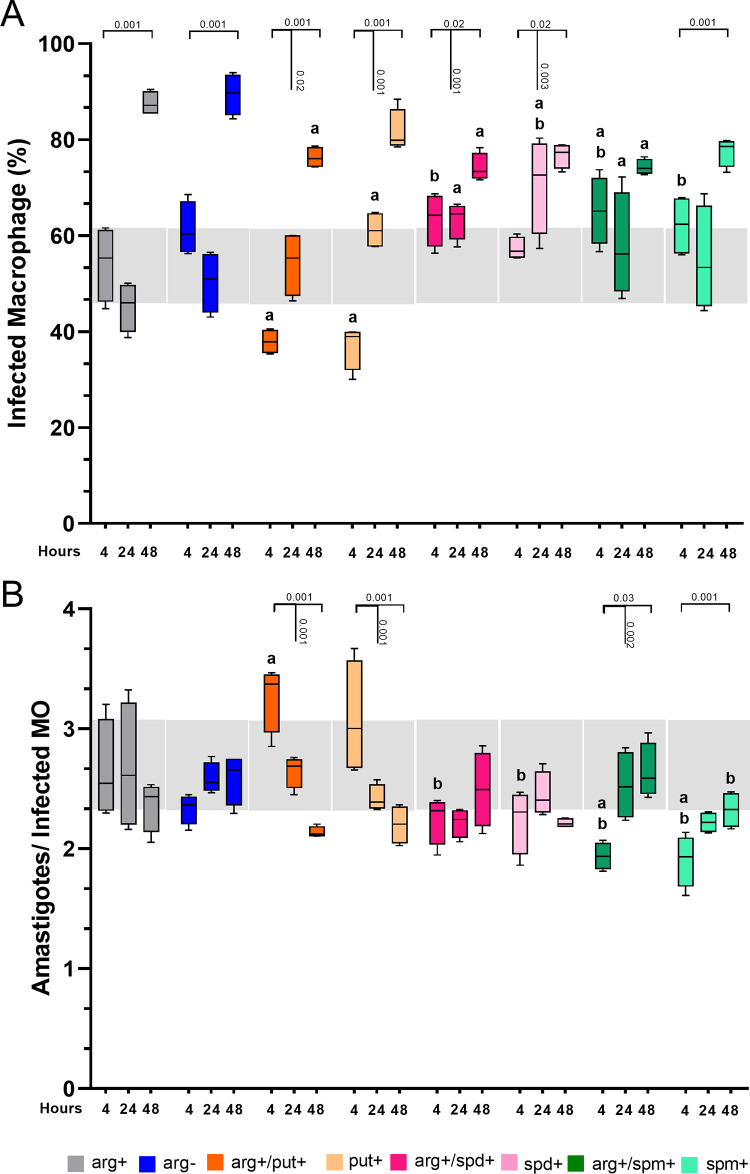
Effect of polyamine supplementation in infection of BALB/c macrophages with *L*. *amazonensis*. Macrophages (2x10^5^) were deprived (arg-) or supplemented (arg+) with L-arginine and/or putrescine (put+), spermidine (spd+), spermine (spm+) concomitant to *L*. *amazonensis* infection (MOI 5:1) for 4h, and after 24 and 48h in complete medium. Cells were stained using Panoptic to determine the percentage of infected macrophage **(A)** and the number of amastigotes per macrophage (B). Each box represents the mean ± S.E.M. of 3 independent experiments (n = 500 macrophages). Statistical analysis using Two-Way ANOVA with mixed-effects, post hoc test Sidak’s multiple comparisons: a, p≤0,05 comparing to arg+; b, p≤0,05 comparing to arg+/put+ and put+.

As shown in Fig 1, the percentage of infected macrophages increased after 48 hours in the presence (arg+) and in the absence (arg-) of L-arginine (p≤0.001) compared to 4 hours (Fig 1A). The number of amastigotes per macrophage was statistically similar among arg+ and arg- at all time points (Fig 1B). Unexpectedly, arg+/put+ and put+ supplementation led to a significantly lower percentage of infected macrophages at 4h compared to arg+ (Fig 1A), which increased at 24 and 48h compared to 4h. However, upon arg+/put+ supplementation, the percentage of infected macrophages reduced at 48h compared to arg+. Curiously, the number of amastigotes per infected macrophage was higher with arg+/put+ and put+ at 4h compared to arg+ but decreased after 24 and 48h.

The supplementation with arg+/spd+ or spd+ increased the percentage of infected macrophages at 24h compared to arg+. (Fig 1A). At 4h of infection, the supplementation with arg+/spd+, spd+, arg+/spm+, or spm+ led to a higher percentage of infected macrophages when compared to arg+/put+ and put+ (Fig 1A). Upon supplementation with arg+/spm+ and spm+, the percentage of infected macrophages was higher than in arg+ at 4 and 24h. In arg+/put+, the number of amastigotes per infected macrophage was higher at 4h compared to arg+, spm+, and spd+ (Fig 1B).

These data indicate that polyamines interfere with the infection in distinct ways. Putrescine supplementation reduced the percentage of infected macrophages, while spermidine and spermine increased.

### 3.2. Polyamines affect the levels *Slc3a2* polyamine transporters

In macrophage infections, the supplementation with arg+/spd+ increased the levels of polyamines transporter *Slc3a2* levels compared to MO-La/arg+ or arg+/put+ at 4h of infection (S2 Fig). On the other hand, the supplementation with arg+/spm+ or spm+ showed lower levels of *Slc3a2* compared to arg+/spd+ at 4h of infection. We observed a reduction in the levels of *Cat1*, *Cat2*, *Slc7a5* at 24h compared to 4h in both infected and uninfected macrophages (S1 and S2 Figs). In infected macrophages, supplementation with spd+ or spm+ increased *Slc7a5* levels at 24h compared to arg+. We did not observe differences in *Slc1a5 or Slc25a15* levels, L-glutamine and L-arginine, and ornithine antiporters, respectively (S1 Fig). Our data indicate that supplementation with L-arginine plus putrescine reduced the expression of *Slc3a2*, once L-arginine plus spermidine supplementation increased *Slc3a2*, indicating different effects of these polyamines in the regulation of *Slc3a2*.

We did not observe the modulation of L-arginine and polyamines transporters *Cat1*, *Cat2*, *Slc3a2*, and *Slc7a5* in conditions of L-arginine deprivation compared to arg+ (S1 Fig). These data indicate that L-arginine *per se* did not affect the expression of genes involved in L-arginine uptake.

Our next aim was to analyze if L-arginine or putrescine could alter the expression of genes related to L-arginine uptake by *Leishmania* in infected macrophages (S3 Fig). We observed increased levels of *Leishmania* transporters *La-aap3 4*.*7* and *La-aap3 5*.*1* under L-arginine deprivation at 4h and of *La-aap3 5*.*1* in put+, suggesting a major impact of L-arginine deprivation and putrescine supplementation in regulating *La-aap3* transporter levels. *Leishmania Arg* and *Nos* levels (La-arg and La-nos, respectively) did not change upon supplementation at 4h and 24h compared to deprived conditions (S3 Fig).

### 3.3. Putrescine modulate *Arg1*, *Arg2* and *Odc1* transcripts

**Fig 2:**
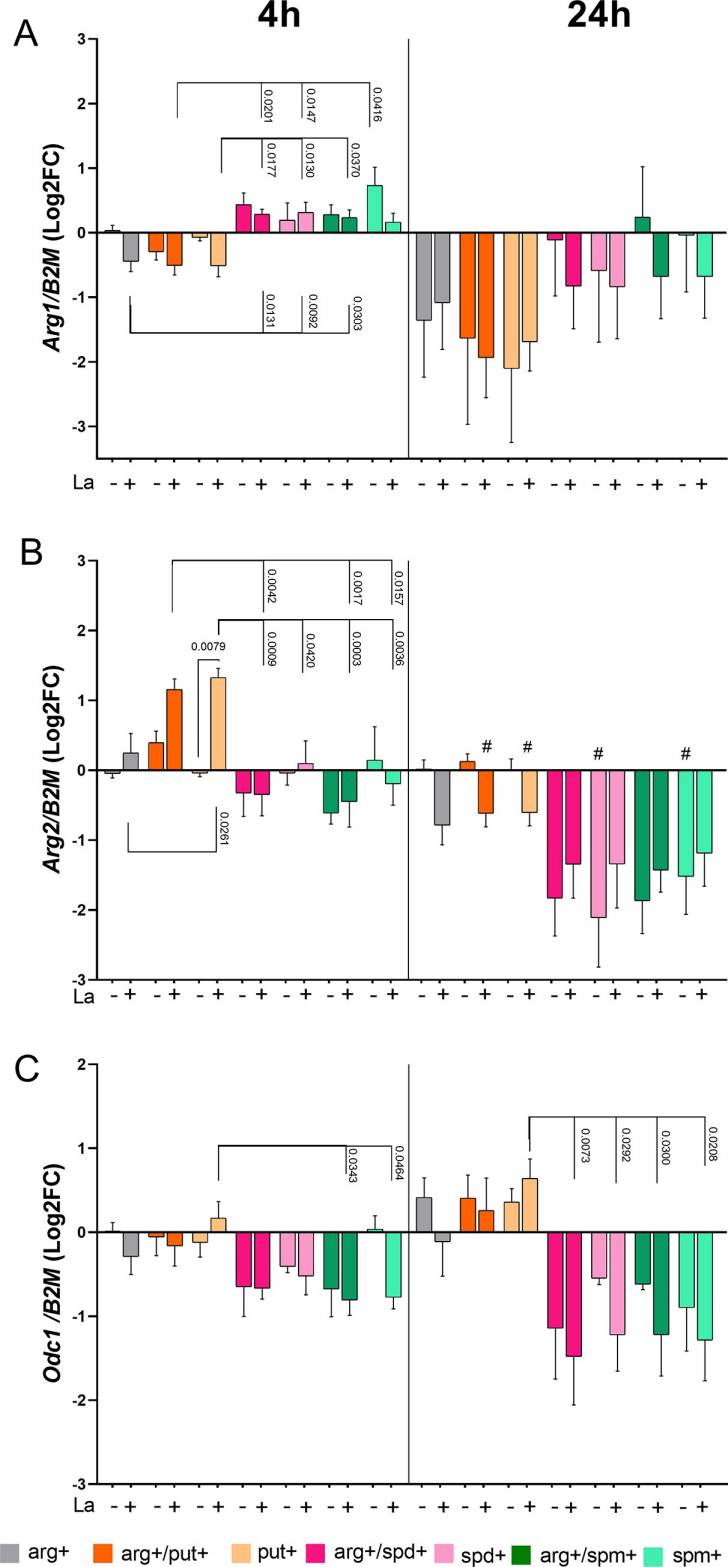
Relative expression of polyamine biosynthesis-related genes in BALB/c macrophages infected or not with *L*. *amazonensis* with polyamine supplementation. Macrophages were supplemented with putrescine (put+), spermidine (spd+), spermine (spm+) with or without L-arginine (arg+) concomitant to *L*. *amazonensis* infection (MOI 5:1) for 4h, and after 24h in complete medium. The RNA was extracted for cDNA conversion and relative quantification of genes *Arg1* **(A)**, *Arg2* **(B)** and *Odc1* **(C)** by RT-qPCR. Data were normalized using the *β-2-microglobulin* gene, and the uninfected macrophage arg+ at 4 h was used as reference in ΔΔCT relative quantification. The bars represent the averages and S.E.M. We performed three independent experiments. Statistical analysis using One-Way ANOVA with mixed-effects, post-hoc test Sidak’s multiple comparisons.

We observed reduced levels of *Arg1* upon putrescine supplementation (arg+/put+ or put+) after 4h of incubation or infection compared with arg+spd+, spd+, and arg+/spm+ (Fig 2). Arg+/put+ or put+ supplementation during 4h of infection led to higher levels of *Arg2* transcripts than arg+. Also, arg+/put+ and put+ supplementation led to higher levels of *Arg2* compared to arg+/spd+, spd+, arg+/spm+, and spm+ (Fig 2B). Put+ supplementation also increased the levels of *Odc1* compared to arg+/spd+ at 4 and 24h of infection (Fig 2C). The supplementation with put increased *SpdS and SpmS* levels at 4 h in infected macrophages compared to uninfected (S4 Fig).

In most conditions, incubations or infections for 24h led to lower levels of *Arg1*and *Arg2* than incubations or infections for 4h (Fig 2).

Under L-arginine deprivation, the levels of *Arg1*, *Arg2*, *Odc1*, *SpdS*, and *SpmS* were similar in uninfected and infected macrophages (S5 Fig). Our data indicate that putrescine supplementation reduced *Arg1* and upregulated *Arg2*, *Odc1*, *SpdS*, and *SpmS* in infected macrophages, while supplementation with spermidine or spermine reverts the gene expression.

### 3.4. Arginine-deprivation and putrescine supplementation increased *Nos2* expression

**Fig 3:**
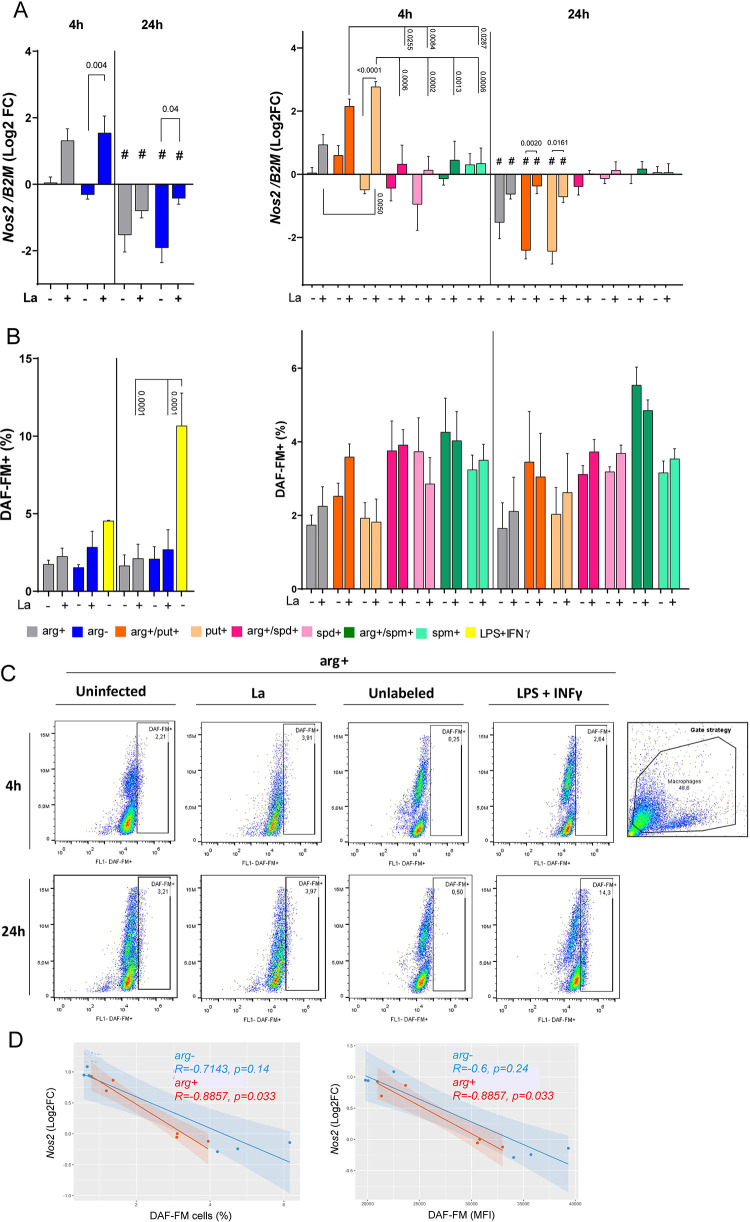
Relative expression of *Nos2* and NO production in BALB/c macrophages infected or not with *L*. *amazonensis* with or without polyamines supplementation. Macrophages (5x10^6^) **(A)** and (1x10^6^) **(B, C)** were supplemented with putrescine (put+), spermidine (spd+), spermine (spm+) with or without L-arginine (arg+) concomitant to *L*. *amazonensis* infection (MOI 5:1) for 4h, and after 24h in complete medium. The RNA was extracted for cDNA conversion and relative quantification of genes *Nos2* **(A)** by RT-qPCR. Data were normalized using the *β-2-microglobulin* gene, and the uninfected macrophage arg+ at 4 h was used as a reference for ΔΔCT relative quantification. The samples were stained with DAF-FM for flow cytometry analysis of DAF-FM+ cells **(B). (C)** Representative dot plot of DAF-FM detection in macrophages uninfected or infected with *L*. *amazonensis* supplemented with arg+ and controls unlabeled or stimulated with LPS plus IFN-γ. **(D)** Correlation of *Nos*2 and NO levels. The bars represent the averages and S.E.M. We performed three independent experiments. Statistical analysis using One-Way ANOVA with mixed-effects, post-hoc test Sidak’s multiple comparisons. **#**: p≤0.05 for the comparison between 4h vs. 24h.

Macrophage *Nos2* levels increased under L-arginine deprivation and also under supplementation with put+ or arg+/put+ at 4h of infection compared to arginine, spermidine, and spermine supplementation (Fig 3A). Also, under putrescine supplementation *Nos2* levels increased at 4h of infection compared to uninfected macrophages (Fig 3A). However, L-arginine deprivation and putrescine supplementation did not lead to a significant increase in the frequency of NOS2 (S6 Fig) and NO production, as stated by the similar frequencies of DAF-FM^+^ cells (Fig 3B) and MFI values (mean of NO production per cell; S6 Fig) in all conditions. In Fig 3C, we show the gating strategy applied to uninfected, infected, or LPS stimulated (positive control) macrophages, unlabeled and labeled with DAF-FM. Curiously, *Nos*2 levels negatively correlated with the percentage of NO-producing cells and median of fluorescence (MFI) of NO production, suggesting that infection with *Leishmania* negatively impacts NO production during arg+ or arg- conditions at the first hours of infection.

Despite that, *Nos2* levels positively correlate with mean of NO production per cell in MO-La/arg+/put+ at 4h (Corr 0.978, p<0.05; S7 Fig), in contrast with arg+ and arg- supplementation group, which shows a negative correlation (Corr -0.964 and -0.907; S7 Fig). The supplementation with spermidine or spermine did not alter *Nos2* levels (Fig 3A). Curiously, the increase in the frequency of DAF-FM^+^ cells during 24h macrophage infection induced by arg+/spm+ negative correlates with *Nos2* levels (Corr -0.925, p < 0.05) and *Nos2* levels negatively correlate with NO production per cell (MFI; Corr -0.929, p < 0.05) (Fig 3B and 3C, S7 Fig).

Our data indicates the augment of *Nos2* expression by L-arginine-deprivation and putrescine supplementation during infection without corresponding with NO production.

### 3.5. Putrescine induce *Mcp1* expression

**Fig 4:**
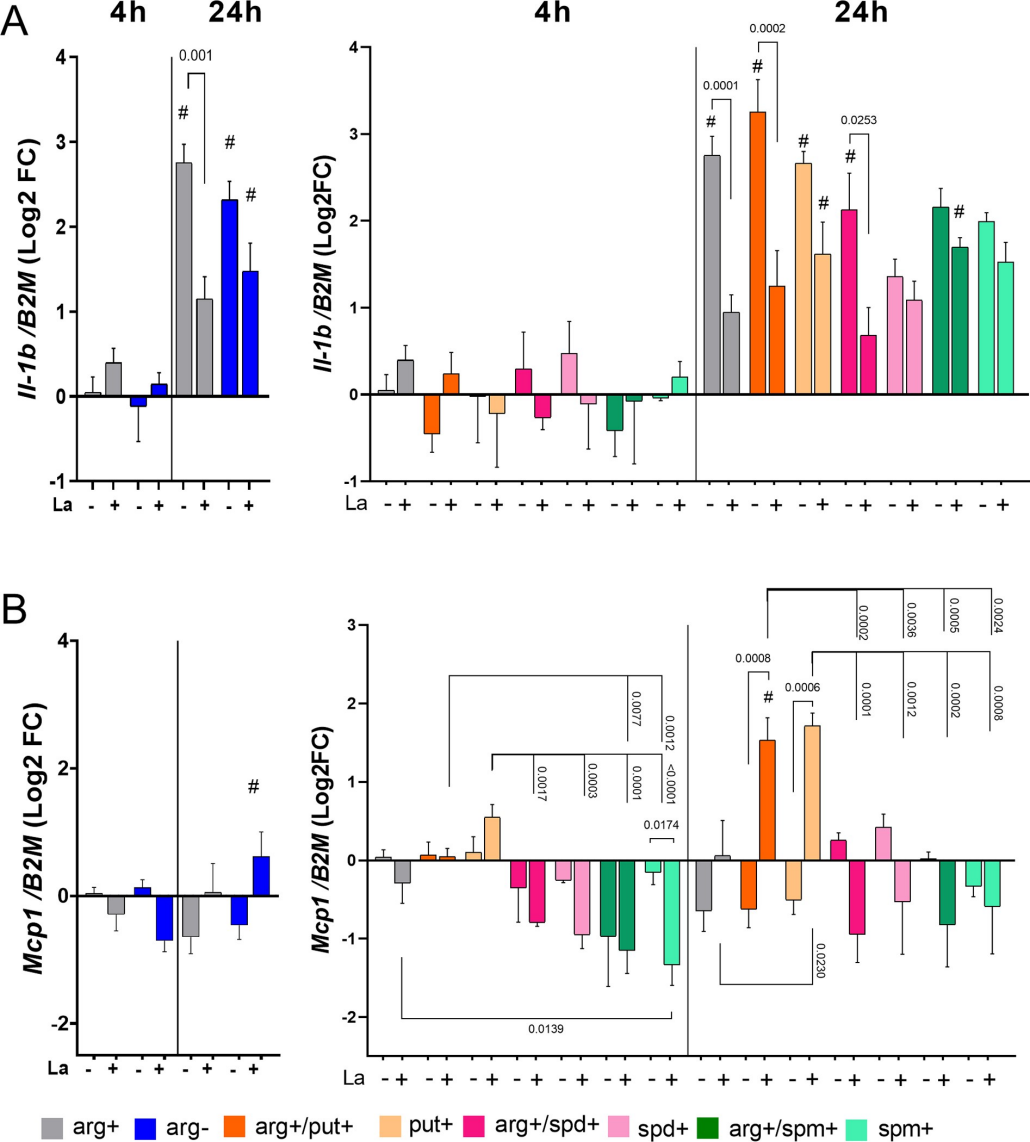
Relative expression of proinflammatory cytokines in BALB/c macrophages infected or not with *L*. *amazonensis* with or without polyamines supplementation. Macrophages were supplemented with putrescine (put+), spermidine (spd+), spermine (spm+) with or without L-arginine (arg+) concomitant or not to *L*. *amazonensis* infection (MOI 5:1) for 4h, and after 24h in complete medium. RNA was extracted for cDNA conversion and relative quantification of genes *Il-1b* **(A) and** *Mcp1* **(B)** by RT-qPCR. Data were normalized using the *β-2-microglobulin* gene, and uninfected macrophage arg+ at 4 h was used as a reference for ΔΔCT relative quantification. The bars represent the averages and S.E.M. We performed three independent experiments. Statistical analysis using One-Way ANOVA with mixed-effects, post-hoc test Sidak’s multiple comparisons. **#**: p≤0.05 for the comparison between 4h vs. 24h.

Next, we analyzed if polyamines could alter the expression of cytokine genes related to the proinflammatory activation of macrophages. At 24h, infected macrophages supplemented with arg+ or arg+/put+ presented an increase in the *Il-1b* levels compared to infected at 4h (Fig 4A). No modifications were observed in *Tnfa* mRNA and TNF protein levels under arginine or polyamines supplementation during *L*. *amazonensis* infection (S8 Fig).

The supplementation with L-arginine increased *Mcp1* levels at 24h of infection compared to uninfected macrophages (Fig 4B). Accordingly, *Mcp1* transcripts are significantly higher after 24h of infection compared to 4h. Macrophages infected for 24h and supplemented with arg+, arg+/put+, or put+ showed an increase in *Mcp1* levels compared to uninfected counterparts (Fig 4B). *Mcp1* levels were higher upon put+ supplementation compared with spm+ or spd+ at 24h of infection. The levels of *Tnfa* transcripts positively correlated with *Mcp1* levels at 24 h of infection in arg+/put+ supplemented macrophages (S8 Fig).

Our data indicate that *Il-1b* and *Mcp1* transcripts can be modulated by infection in the presence of L-arginine and putrescine. More specifically, *Mcp1* levels increased upon infection in the presence of arg+/put+ and put+, while they reduced upon spermidine and spermine supplementation.

**Fig 5:**
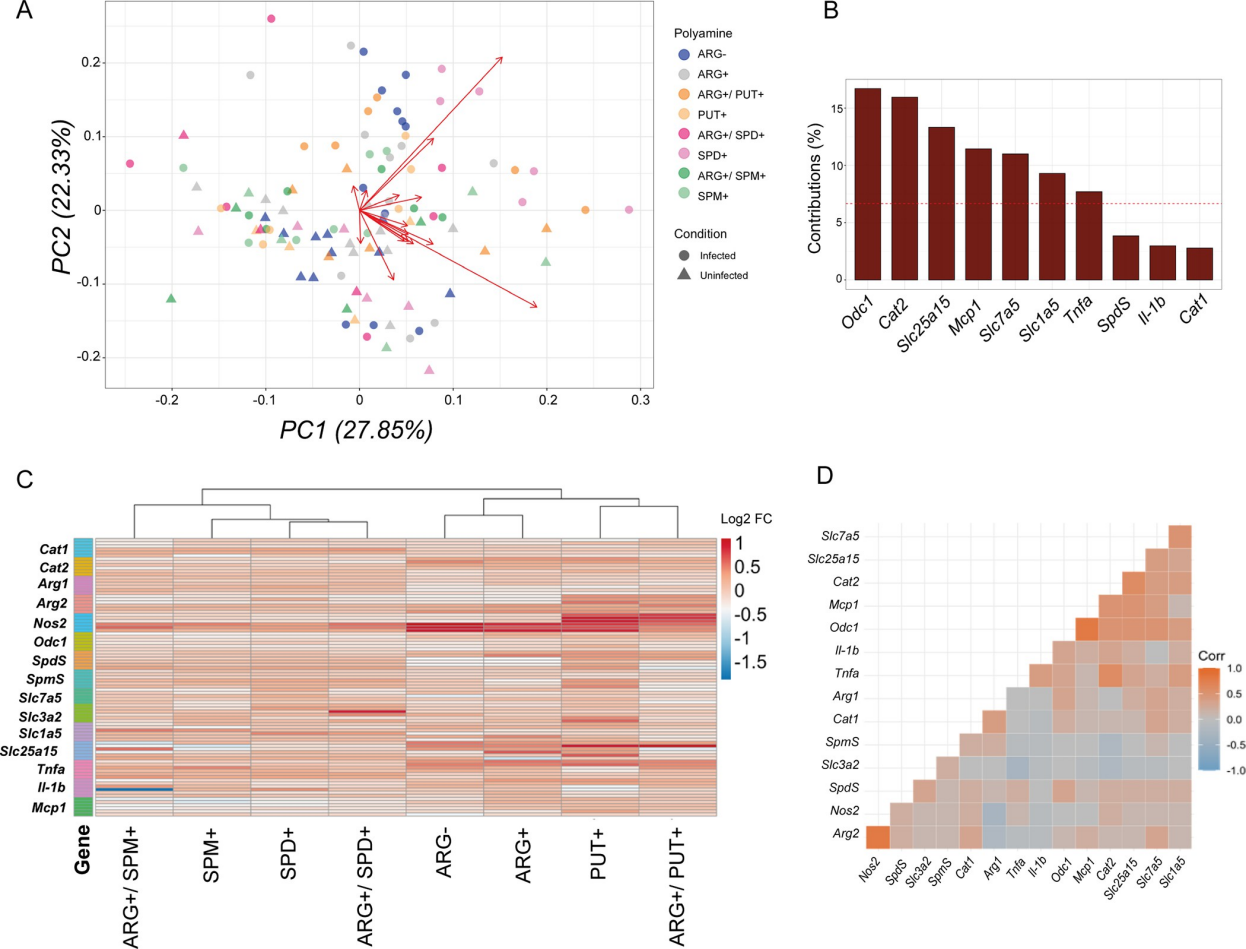
Correlation and heat map analysis. **(A)** PCA analysis of gene expression variance in all conditions analyzed after 4h in infected and non-infected macrophages and **(B)** percentage of contributions of each gene to the Principal Component 1 (PC1). **(C)** The Heat Map analysis of the Log2-fold change of genes upregulated (red) and downregulated (blue) and **(D)** gene expression correlation matrix (Spearman correlation) showed positive (orange) and negative (blue) associations between gene expression from data of macrophages supplemented with putrescine (put+), spermidine (spd+), spermine (spm+) with or without L-arginine (arg+) concomitant or not to *L*. *amazonensis* infection for 4h.

The PCA (Fig 5A) and heat map (Fig 5C) represent gene expression levels in all conditions analyzed after 4h of infection. Unexpectedly, the levels of *Odc1*, *Cat2*, and *Slc25a15* at 4h, and *SpmS*, *Cat1*, and *Nos2* contribute to sample dispersion in the PCA. The heat map allowed us to visualize the response obtained upon putrescine supplementation with or without L-arginine concomitant to infection, characterized by induction of *Nos2* and *Arg2*, and the response to spermidine supplementation at 4h, with downregulation of *Mcp-1* and *IL1b*. In addition, putrescine maintained its clusterization patterns in relation to spermidine and spermine, suggesting that the polyamines effect over the gene transcription is more evident in a later stage of infection or treatment. Fig 5D shows correlations between the expression of genes after 4h of infection. At 4h of infection, stronger correlations were observed between *Arg2* and *Nos2* and between *Odc1* and *Mcp1*.

**Fig 6:**
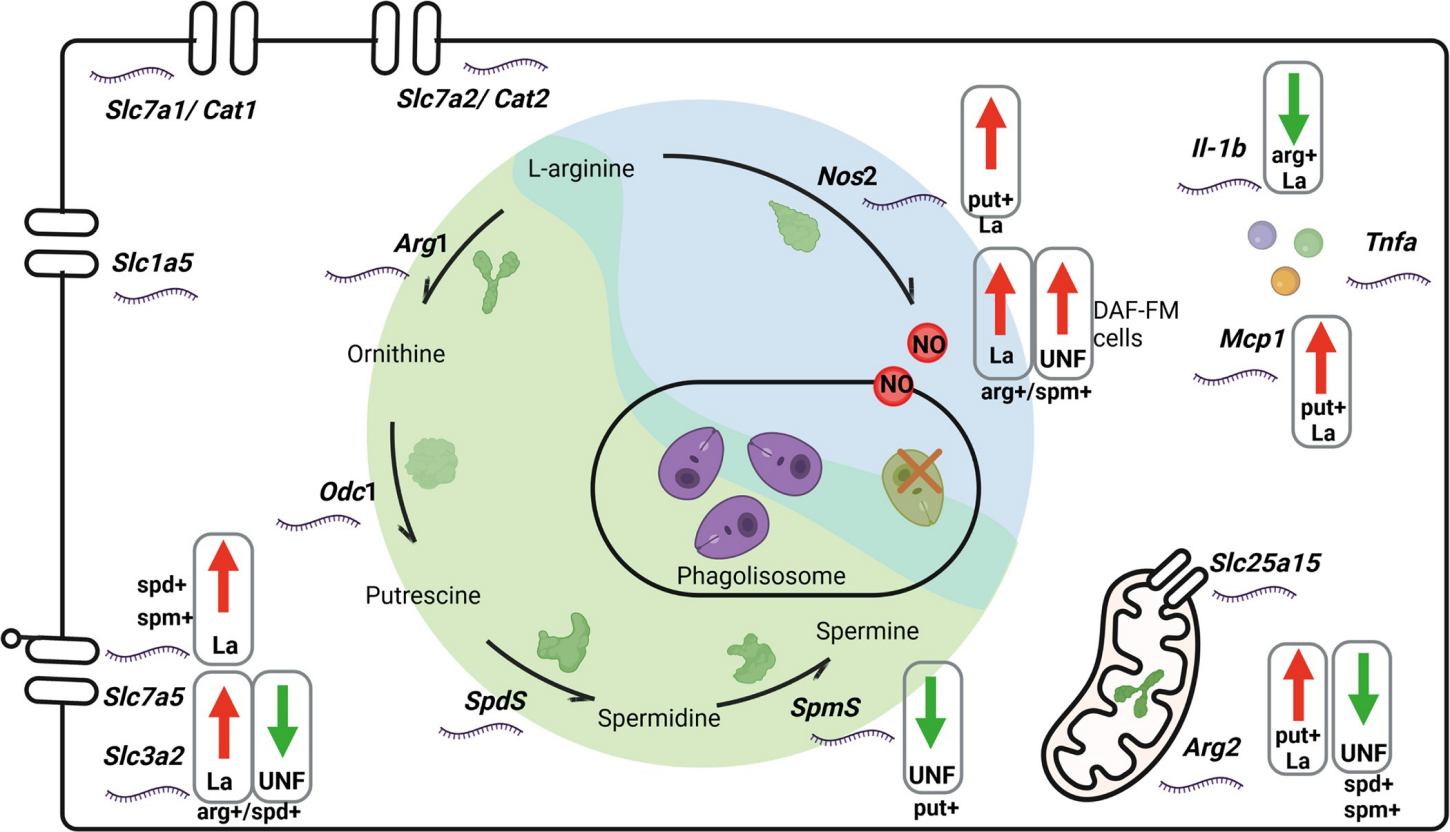
Effects of L-arginine and polyamines supplementation in gene expression in BALB/c macrophage during infection with *L*. *amazonensis*. Genes modulated by putrescine, spermidine, spermine, or L-arginine. The L-glutamine transporter SLC1A5 is shown in the mitochondrial membrane. SLC25A15 performs the L-citrulline and L-ornithine antiport between the mitochondria and cytosol. The enzymes from polyamines biosynthesis: arginase 1 (ARG1) converts L-arginine into ornithine, which is subsequently converted by ornithine decarboxylase 1 (ODC1) into putrescine. Putrescine is converted by spermidine synthase (SPDS) into spermidine; spermidine is converted by spermine synthase (SPMS) into spermine. The Nitric oxide 2 (NOS2) enzyme uses L-arginine to produce nitric oxide (NO) and citrulline. Also, mitochondrial arginase 2 (ARG2) uses L-arginine to produce ornithine. E, extracellular environment. The L-arginine transporters CAT1 and CAT2 and the heterodimeric amino acid and polyamines transporter SLC3A2/SLC7A5 are shown in the plasmatic membrane. The amino acid and polyamines transporters can also mediate transport in the phagolysosome membrane. Created with Biorender.com.

Fig 6 shows a comprehensive panel of our findings, integrating data on the abundance of the transcripts of transporters and enzymes with information on supplementation and infection.

## 4. Discussion

In the current study, we focused on analyzing macrophage infection and the transcript levels of genes related to L-arginine/polyamines transport and metabolism in BALB/c macrophages upon deprivation or supplementation with L-arginine, putrescine, spermidine, and spermine during infection with *L*. *amazonensis*. L-arginine availability is implicated in the outcome of *Leishmania* infection since the competition for this amino acid by the host and parasite arginase and NOS2 affect NO production and consequently parasite killing [43, 46–49].

Unexpectedly, L-arginine deprivation increased *Nos*2 levels at 4 h of infection without affecting NO production. Curiously, we also showed that deprivation of L-arginine during *L*. *amazonensis* infection of BALB/c macrophages did not modulate the expression of the genes *Arg1*, *Arg2*, *Odc1*, *SpdS*, and *SpmS*, related to polyamines production. We have previously reported increased levels of L-arginine, ornithine, putrescine, spermine, and glutamine in metabolomic data from BALB/c and C57BL/6 macrophages after 4h of infection with *L*. *amazonensis* [39, 50]. Infection of BALB/c macrophages with *L*. *amazonensis* knockout for arginase led to the accumulation of L-arginine during infection, while proline, ornithine, and putrescine were diminished relative to infections with wild-type parasites [39, 50]. Intriguingly, we showed that the deprivation of L-arginine did not alter the infection of macrophages. We hypothesize that the internal pool of L-arginine can supply for polyamine production in situations of L-arginine deprivation during infection. Besides, we did not observe any effect of deprivation in *Cat1*, *Cat2*, *Slc3a2*, and *Slc7a5* levels (S1 and S2 Figs). Interestingly, BALB/c macrophages knockout for *Cat2* present reduced transport of L-arginine during stimulation with IFN-γ plus LPS or IL-4 plus IL-10, without modifications in NOS2 or ARG1 levels [51].

The parasite arginase can consume L-arginine from the host, as described for amastigote forms consuming nutrients from phagolysosome [52]. Parasite L-arginine transporter *La-aap3* was upregulated during L-arginine deprivation and putrescine supplementation, suggesting that the parasite senses the change in the L-arginine availability in the first hours of infection. We did not find modifications on parasite arginase (*La-arg*) or Nos-like (*La-nos*) levels. We also hypothesized that polyamines might be transported from host to parasite, affecting the host’s internal pool and transport. Polyamines availability can interfere with the levels of L-arginine transporters (CAT1/2) and L-arginine metabolism by enzymes ARG1 and ARG2, as well as in the enzymes of polyamine pathway ODC, SPDS, and SPMS [6]. Also, polyamine uptake depends on the expression of host polyamine transporter SLC3A2/SLC7A5 [19–22]. Indeed, we observed that after putrescine, spermidine, or spermine supplementation, the levels of *Cat1*, *Cat2*, *Slc3a2*, and *Slc7a5* suffered some alterations, suggesting that infection and polyamines can influence transporter genes’ transcription.

*L*. *amazonensis* infection causes severe cutaneous lesions in BALB/c mice but only moderate lesions in C57BL/6 [48, 49, 53, 54]. Similarly, BALB/c mice are susceptible to *L*. *major* infection, increasing IL-4 and IL-10 production, characteristic of Th2 response. In contrast, C57BL/6 is a resistant model to *L*. *major*, due to development of a Th1-response [55–58]. Despite that, IL-4 and IL-10 knockout mice have impaired IL12 production and Th1 response and are not able to resolve infection by *L*. *amazonensis* or *L*. *major* [59–61].

The recognition of parasites by macrophages in the early phase of infection can affect infection control. For example, TLR2-deficient mice show reduced *L*. *amazonensis* parasite loads. However, *in vitro* infection of macrophages deficient in TLR2, TLR4, and MyD88 by *L*. *amazonensis* is higher than wild-type counterparts [62]. TLR4 and MyD88 deficient macrophages infected with *L*. *amazonensis* exhibited reduced levels of *Cat1*, *Cat2*, *Arg1*, *and Nos2* and increased levels of miRNA let-7e, which inhibited *Nos2* and NO production [62, 63].

We showed that the supplementation with putrescine increased *Nos2* levels without modulating NO production. The production of NO depends on the activation of NOS2 and the availability of L-arginine, NADPH, biopterin, and O_2_ [64]. NO levels are limited in *L*. *major* skin lesions, probably by the low O_2_ pressure in the micromilieu [65]. However, we have already reported NO production in *L*. *amazonensis* infected C57BL/6 macrophages [39, 50, 66]. NO-producing cells were observed in L-arginine plus spermine supplementation in *L*. *amazonensis*-infected macrophages without *Nos2* modulation. Curiously, another group reported that spermidine reduced the expression of *Nos*2 in LPS-stimulated macrophages [67]. It was previously shown that BALB/c macrophage knockout for *Nos2* did not differ in the expression of *Cat1* and *Cat2* and transported L-arginine during stimulation with IFN-γ plus LPS or IL-4 plus IL-10 [51].

Putrescine supplementation reduced the percentage of infected macrophages, contrarily to what we observed upon spermidine and spermine supplementation. These results suggest that putrescine effects differ from those of spermine and spermidine, improving macrophage activation and leishmanicidal capacity. In contrast to our expectations, we did not observe a correlation between a reduction in *Arg1* and an increase in *Nos2*, reflecting in changes in NO production upon putrescine supplementation during infection. This lack of correlation can be due to increased levels of *Arg2* or substrate availability to NO production after 4h. Some studies showed that increased host ARG1 cause L-arginine depletion inside the cell, reducing the NOS2/NO leishmanicidal activity on infected macrophages, inflammation, and activation of T cells [16, 68].

In contrast, lower numbers of *L*. *major* were reported close to ARG1^+^-cells. Also, the lack of *Arg1* in hematopoietic cells from C57BL/6 mice does not coordinate the resolution of inflammation and tissue repair [17, 69]. In *L*. *amazonensis* skin lesions in C57BL/6 mice, CD11b^+^-cells expressed ARG1 and NOS2, and the lack of IFN-γ reduced NOS2 levels [70]. In *L*. *major* skin lesions, the lack of *Arg*1 was not compensated by increased *Arg*2 levels [17]. Despite the lack of polyamine biosynthetic pathways in mitochondria, we can speculate that an increase in *Arg*2 induced by putrescine can guide the use of L-arginine by ARG2-increasing ornithine, which could be converted to citrulline inside mitochondria or proline and glutamine in the cytoplasm [71–73]. The increased levels of ornithine, proline, and glutamine found in the *L*. *amazonensis* infected BALB/c macrophages can support glutamate production, interfering in the metabolic and redox state of macrophages during activation [39, 50, 71, 73–75].

Spermine and spermidine play a role in protecting cells from reactive oxygen species (ROS). Spermidine is known to reduce the expression of ROS in LPS-stimulated macrophages [67]. Polyamines, specially spermine, can indirectly mediate Ca^2+^ transport or function on mitochondrial respiration, stimulating succinate dehydrogenase activity and increasing mitochondrial reactive oxygen species (mtROS) production [76–78]. We have already reported higher levels of glutathione and trypanothione in BALB/c and C57BL/6 macrophages after 4h of infection with *L*. *amazonensis* [39, 50, 66, 79]. *Leishmania* can use glutathione and spermidine to produce glutathionylspermidine and trypanothione, an essential molecule to protect the parasite against the mammalian host defense [80, 81]. Spermine induces superoxide dismutase synthesis and can prevent oxidative damage [82, 83]. The dysregulation of antioxidant activity leads to ROS accumulation and affects mitochondrial integrity [84]. Spermine negatively regulates macrophage activation via polyamines catabolism mediated by acetylation via N1-spermidine/spermine acetyltransferase (SSAT) [85, 86].

On the other hand, the accumulation of polyamines can cross-regulate metabolic-related genes and inflammation during infection. Also, it was shown that *SpmS* knockout causes the accumulation of spermidine and an increase of aldehyde and hydrogen peroxide (H_2_O_2_), leading to lysosomal dysfunction and oxidative stress [83]. Spermine was also shown to inhibit the translation of NOS2 in macrophages, reducing NO production [55]. IL-4 stimulation induces ODC, increasing putrescine production in murine macrophages, and the inhibition of ODC with difluomethylornithine (DFMO) reduces putrescine content but not spermidine and spermine [87, 88]. In this context, macrophages stimulated with IL-4 display increased putrescine levels [42, 43].

Regarding the expression of genes related to macrophage polarization, putrescine supplementation during infection increased the expression of *Nos2* and *Mcp1* independently of L-arginine supplementation. Previous studies showed that putrescine and spermine increase MCP-1 and TNF-α in mixed glial culture [89]. Spermidine reduces the secretion of TNF-α and IL-1β in LPS-stimulated RAW 264.7 macrophages [67] and MCP-1 secretion in THP-1-macrophages treated with IFN-γ [90]. IL-1β induces NOS2 and NO production and resistance to infection in C57BL/6 BMDM infected with *L*. *amazonensis* [91], and transcriptome data showed downregulation of *Il1b* in *L*. *amazonensis* infected BALB/c-BMDM [92]. In *L*. *amazonensis* skin lesions on C57BL/6 mice, the lack of CCR2 (receptor for MCP-1) CD11b^+^-cells showed lower ARG1 and NOS2 and a reduction in parasite load [70]. MCP-1 increases phagocytosis of bacteria *Escherichia coli* in BMDMs and apoptotic-neutrophils by murine macrophages and increases respiratory burst and release of superoxide anion, implicating a production of MCP-1 to the phagocytic capacity of macrophages [93–96].

The polarization to M2 macrophages during *L*. *amazonensis* and *L*. *major* infection *in vitro* is associated with parasite growth [97]. M2 macrophages affect the severity of cutaneous disease by regulating chronic inflammation, parasite internalization, and elimination [98–102]. Interestingly, in the lesions of diffuse cutaneous leishmaniasis (DCL) the levels of *Arg1*, *Cat2*, and *SpmS* mRNAs were upregulated in relation to localized cutaneous leishmaniasis (LCL) or mucocutaneous leishmaniasis (MCL) patients, correlating with higher levels of ornithine and spermidine, but not of arginine [101, 102]. Also, *Arg1* levels negatively correlated with parasite load in LCL and DCL lesions. The levels of IL-4 and IL-10 mRNAs were higher in relation to TNF in lesions of DCL [101, 102]. Whether the increased expression of *Arg2*, *Nos2*, *SpmS*, and *Mcp-1* in putrescine supplementation in infected macrophages is associated with macrophage polarization during infection warrants further investigation.

Data from the literature highlight the importance of L-arginine and polyamines in several processes related to *Leishmania in vitro* and *in vivo* infection. In this work, we analyzed the importance of these molecules in *L*. *amazonensis* infection in BALB/c-macrophages. One of our main findings was that infection of macrophages was lower after putrescine supplementation than after L-arginine, spermidine, and spermine, and putrescine modulated the expression of L-arginine-metabolism related-genes on BALB/c macrophages. We hope these results stimulate other studies on the importance of polyamines in macrophage metabolism and *Leishmania* infection.

## Supporting information

S1 Fig Relative expression of cationic and neutral transporters in BALB/c macrophages infected or not with *L*. *amazonensis* with or without polyamines supplementation. Macrophages were deprived of L-arginine (arg-) or supplemented with L-arginine (arg+) concomitant or not to *L*. *amazonensis* infection (MOI 5:1) for 4h and after 24h in complete medium. RNA was extracted for cDNA conversion and quantification of *Cat*1 **(A)**, *Cat2* **(B),** *Slc1a5* **(C),** and *Slc25a15***(D)** transcripts by RT-qPCR. Data were normalized using the *β-2-microglobulin* gene, and the uninfected macrophage arg+ at 4 h was used as a reference for ΔΔCT relative quantification. The bars represent the averages and S.E.M. We performed three independent experiments. Statistical analysis using One-Way ANOVA was indicated in the bars. **#**: p≤0.05 for the comparison between 4h vs. 24h.
(TIF)

S2 Fig Relative expression of polyamine heterodimer transporters in uninfected and infected BALB/c macrophages. The macrophages (5x10^6^) were supplemented with L-arginine (arg+) and/or putrescine (put+), spermidine (spd+), spermine (spm+), simultaneously to *L*. *amazonensis* infection, maintained in the MOI proportion of 5:1 for 4h and, after, to more 24h in complete medium. After 4 and 24h, the RNA was extracted for cDNA conversion and relative quantification of genes *Slc3a2* **(A)** and *Slc7a5* **(B)** by RT-qPCR. The data were normalized using the *β-2-microglobulin* gene. The uninfected macrophages supplemented with arg+ at 4h were used as a control in ΔΔcT calculus. The bars represent the averages and S.E.M of the values. One-Way ANOVA analysis indicates less or equal values or symbols above the bars. #: p≤0,05 for comparing 4h vs. 24h.
(TIF)

S3 Fig Relative expression of parasite transporters and metabolism of L-arginine in BALB/c macrophages infected or not with *L*. *amazonensis* with or without putrescine supplementation. Macrophages were deprived of L-arginine (arg-) or supplemented with L-arginine (arg+), putrescine (put+), with or without L-arginine (arg+) concomitant or not to *L*. *amazonensis* infection (MOI 5:1) for 4h, and after 24h in complete medium. RNA was extracted for cDNA conversion and quantification of La-aap3 4.7 **(A)**, La-aap3 5.1 **(B)**, *La-arg* **(C),** *and La-nos* **(D)** transcripts by RT-qPCR. Data were normalized using the *β-2-microglobulin* gene, and the infected macrophage arg+ at 4 h was used as a reference for ΔΔCT relative quantification. The bars represent the averages and S.E.M. We performed three independent experiments. Statistical analysis using One-Way ANOVA was indicated in the bars. **#**: p≤0.05 for the comparison between 4h vs. 24h.
(TIF)

S4 Fig Relative expression of polyamine biosynthesis-related genes in BALB/c macrophages infected or not with *L*. *amazonensis* with polyamine supplementation. Macrophages were supplemented with putrescine (put+), spermidine (spd+), spermine (spm+) with or without L-arginine (arg+) concomitant to *L*. *amazonensis* infection (MOI 5:1) for 4h, and after 24h in complete medium. The RNA was extracted for cDNA conversion and relative quantification of genes *SpdS* **(A)** and *SpmS* **(B)** by RT-qPCR. Data were normalized using the *β-2-microglobulin* gene, and the uninfected macrophage arg+ at 4 h was used as a reference in ΔΔCT relative quantification. The bars represent the averages and S.E.M. We performed three independent experiments. Statistical analysis using One-Way ANOVA with mixed-effects, post-hoc test Sidak’s multiple comparisons.
(TIF)

S5 Fig Relative expression of L-arginine metabolism related genes and NO production in BALB/c macrophages infected or not with *L*. *amazonensis* in conditions of L-arginine deprivation or supplementation. Macrophages were deprived of L-arginine (arg-) or supplemented with L-arginine (arg+) concomitant to *L*. *amazonensis* infection (MOI 5:1) for 4h and 24h. Relative quantification of *Arg1* **(A)**, *Arg2* **(B)**, *Odc1* **(C),** *SpdS* **(D),** and *SpmS* **(E)** transcripts was performed by RT-qPCR. Data were normalized using the *β-2-microglobulin* gene, and uninfected macrophage arg+ at 4 h was used as a reference for ΔΔCT relative quantification. The bars represent the averages and S.E.M. We performed three independent experiments. Statistical analysis using One-Way ANOVA with mixed-effects, post hoc test Sidak’s multiple comparisons. **#**: p≤0.05 for the comparison between 4h vs. 24h.
(TIF)

S6 Fig Expression of NOS2 and NO production in BALB/c macrophages infected or not with *L*. *amazonensis* with or without polyamines supplementation. Macrophages (3x10^6^) (A) and (1x10^6^) (A-B) were supplemented with putrescine (put+), spermidine (spd+), spermine (spm+) with or without L-arginine (arg+) concomitant to *L*. *amazonensis* infection (MOI 5:1) or stimulated with LPS for 4h, and after 24h in complete medium. The samples were stained with anti-NOS2 for flow cytometry analysis of intracellular levels of NOS2 **(A)** or DAF-FM for flow cytometry analysis of DAF-FM MFI **(B)**. The bars represent the averages and S.E.M. We performed three independent experiments. Statistical analysis using One-Way ANOVA with mixed-effects, post-hoc test Sidak’s multiple comparisons.
(TIF)

S7 Fig Correlation matrix. The correlation analysis using ggpairs plot matrix of the percentage of infected macrophage (MO.La), amastigote per infected macrophage (Amas.MO), percentage of NO producing cells (DAF-FM.pos), Mean of fluorescence intensity of NO production (MFI) and *Nos2* expression. The correlation plots were generated by GGally (1.5.0) or ggstatplot (0.9.0), the proper correlation tests were conducted with an established p of 0.05.
(TIF)

S8 Fig Relative expression of proinflammatory cytokines in BALB/c macrophages infected or not with *L*. *amazonensis* with or without polyamines supplementation. Macrophages were supplemented with putrescine (put+), spermidine (spd+), spermine (spm+) with or without L-arginine (arg+) concomitant or not to *L*. *amazonensis* infection (MOI 5:1) or stimulated with LPS for 4h, and after 24h in complete medium. RNA was extracted for cDNA conversion and relative quantification of genes *Tnf* **(A)** by RT-qPCR. Data were normalized using the *β-2-microglobulin* gene, and uninfected macrophage arg+ at 4h was used as a reference for DDCT relative quantification. The samples were stained with APC-anti-IL1B **(B)** or PE-anti-TNF **(C)** for flow cytometry analysis of intracellular levels of IL1B or TNF.
(TIF)
