# Supplementary figures and images for: Putrescine supplementation shifts macrophage L-arginine metabolism related-genes reducing Leishmania amazonensis infection
Source: PLoS One. 2023 Mar 31;18(3):e0283696. doi: 10.1371/journal.pone.0283696 (PMC10065296; doi:10.1371/journal.pone.0283696)

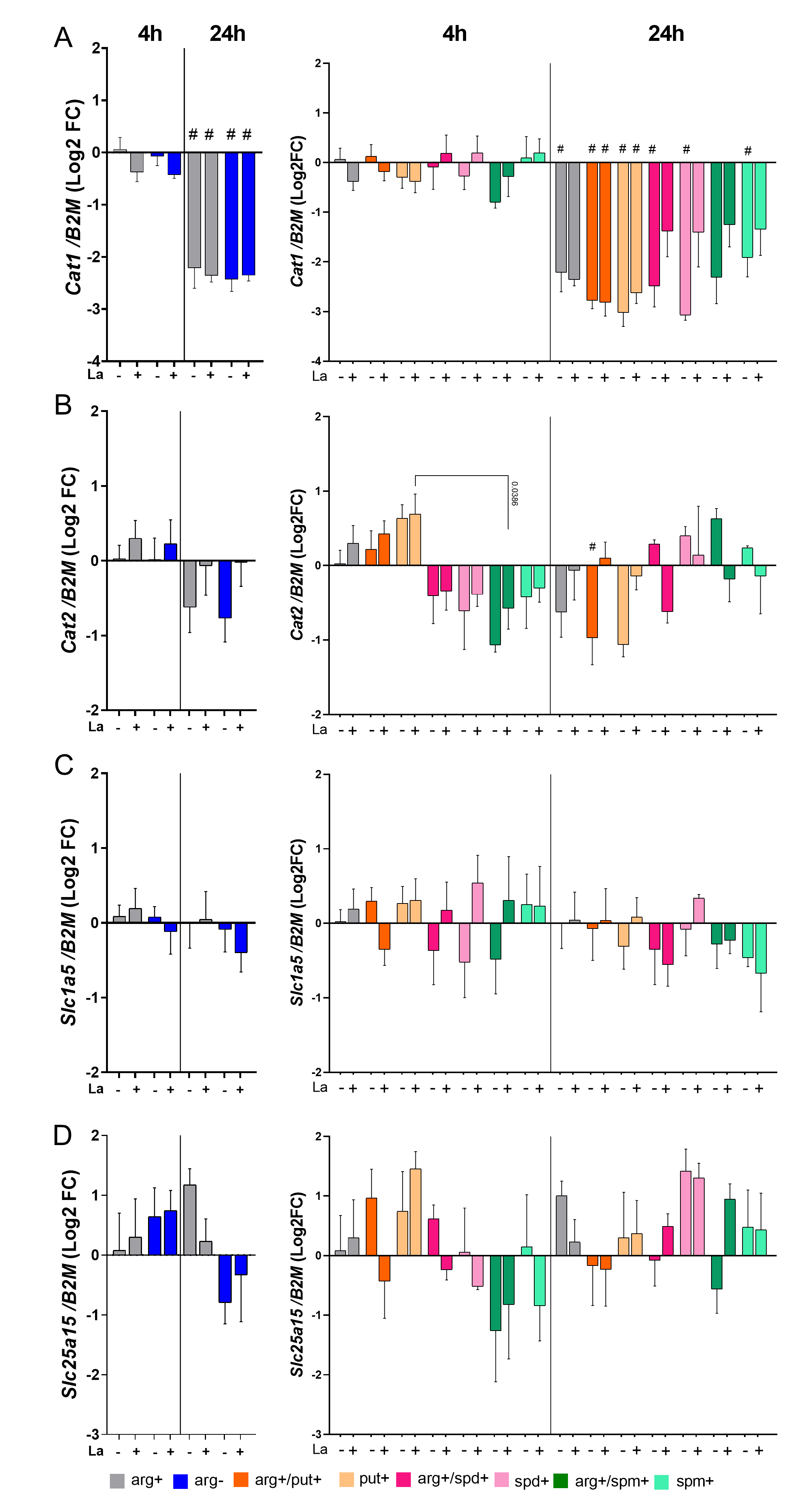

Supplement: S1 Fig — Macrophages were deprived of L-arginine (arg-) or supplemented with L-arginine (arg+) concomitant or not to L. amazonensis infection (MOI 5:1) for 4h and after 24h in complete medium. RNA was extracted for cDNA conversion and quantification of Cat1 (A), Cat2 (B), Slc1a5 (C), and Slc25a15(D) transcripts by RT-qPCR. Data were normalized using the β-2-microglobulin gene, and the uninfected macrophage arg+ at 4 h was used as a reference for ΔΔCT relative quantification. The bars represent the averages and S.E.M. We performed three independent experiments. Statistical analysis using One-Way ANOVA was indicated in the bars. #: p≤0.05 for the comparison between 4h vs. 24h. (TIF) [file pone.0283696.s001.tif]

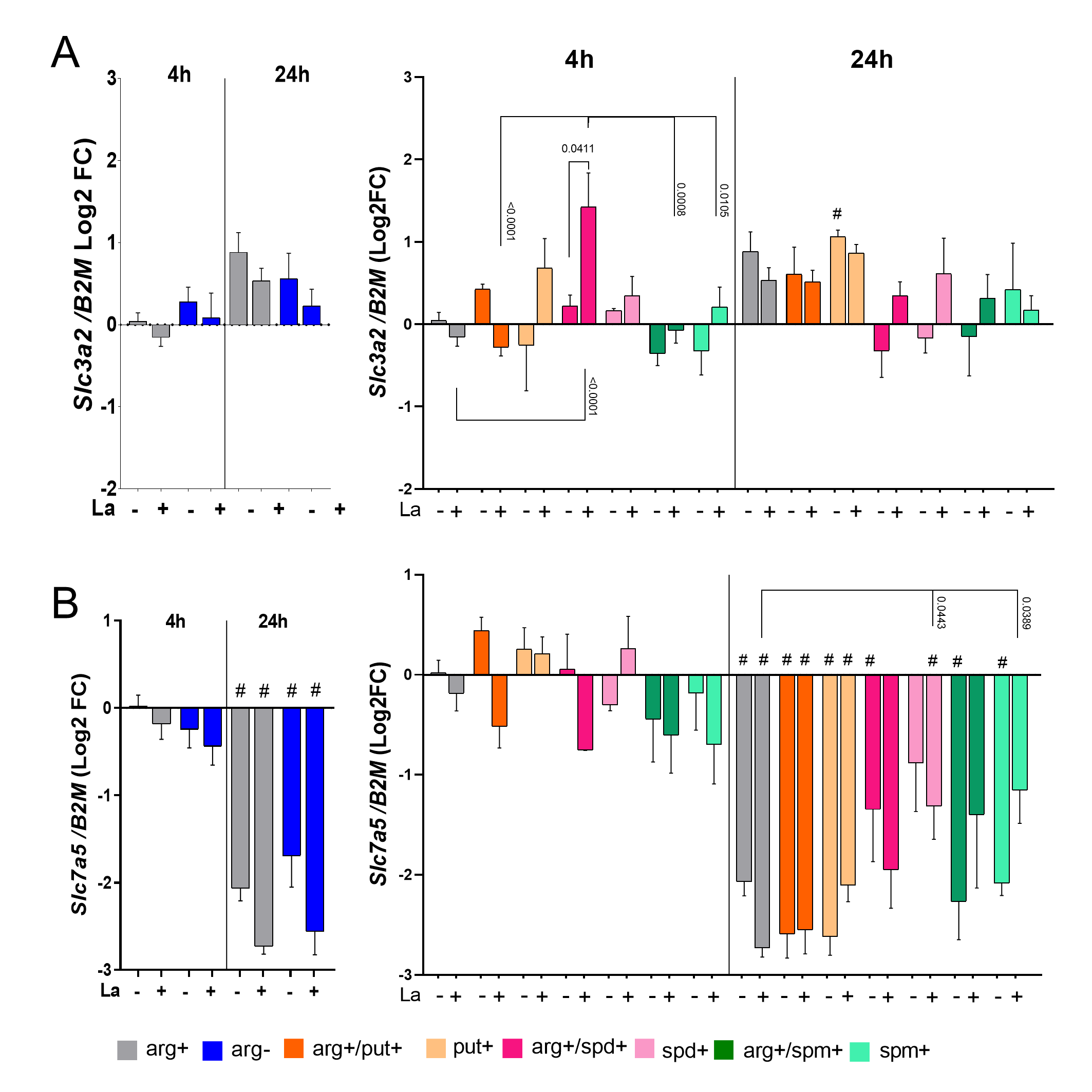

Supplement: S2 Fig — The macrophages (5x10^6^) were supplemented with L-arginine (arg+) and/or putrescine (put+), spermidine (spd+), spermine (spm+), simultaneously to L. amazonensis infection, maintained in the MOI proportion of 5:1 for 4h and, after, to more 24h in complete medium. After 4 and 24h, the RNA was extracted for cDNA conversion and relative quantification of genes Slc3a2 (A) and Slc7a5 (B) by RT-qPCR. The data were normalized using the β-2-microglobulin gene. The uninfected macrophages supplemented with arg+ at 4h were used as a control in ΔΔcT calculus. The bars represent the averages and S.E.M of the values. One-Way ANOVA analysis indicates less or equal values or symbols above the bars. #: p≤0,05 for comparing 4h vs. 24h. (TIF) [file pone.0283696.s002.tif]

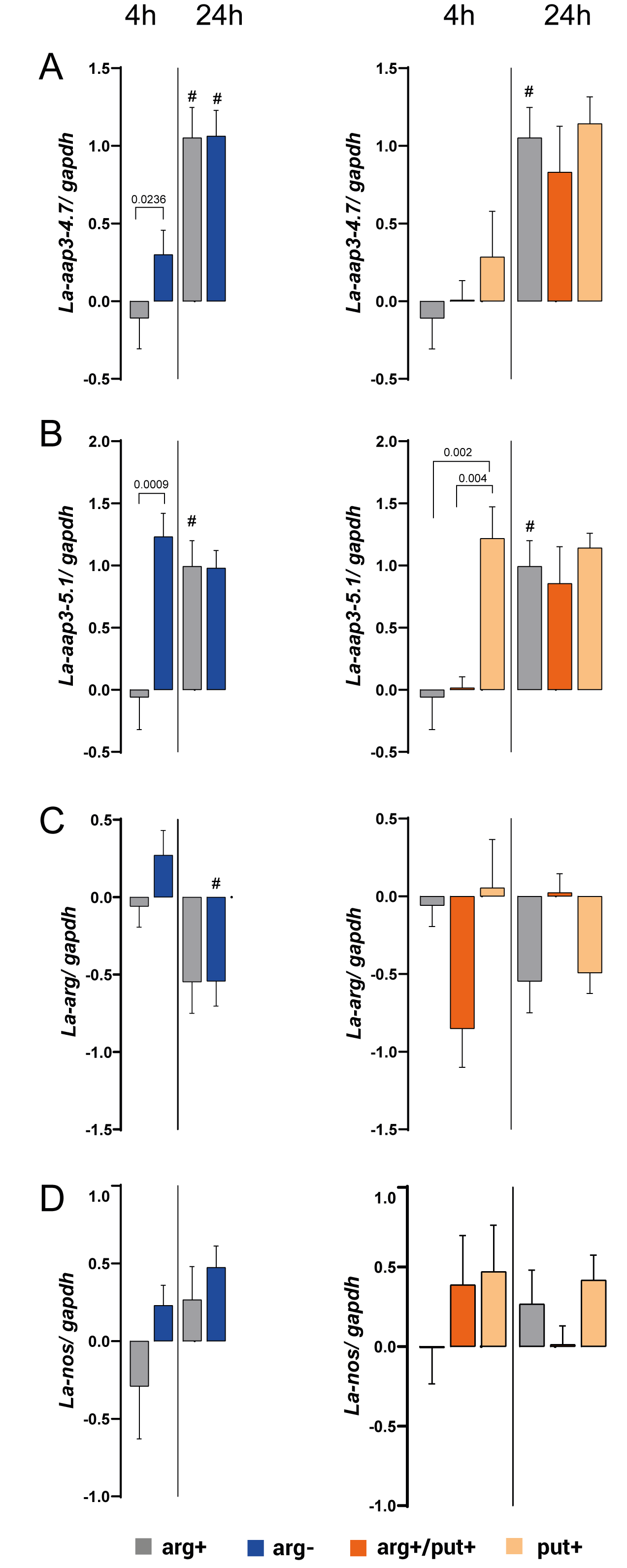

Supplement: S3 Fig — Macrophages were deprived of L-arginine (arg-) or supplemented with L-arginine (arg+), putrescine (put+), with or without L-arginine (arg+) concomitant or not to L. amazonensis infection (MOI 5:1) for 4h, and after 24h in complete medium. RNA was extracted for cDNA conversion and quantification of La-aap3 4.7 (A), La-aap3 5.1 (B), La-arg (C), and La-nos (D) transcripts by RT-qPCR. Data were normalized using the β-2-microglobulin gene, and the infected macrophage arg+ at 4 h was used as a reference for ΔΔCT relative quantification. The bars represent the averages and S.E.M. We performed three independent experiments. Statistical analysis using One-Way ANOVA was indicated in the bars. #: p≤0.05 for the comparison between 4h vs. 24h. (TIF) [file pone.0283696.s003.tif]

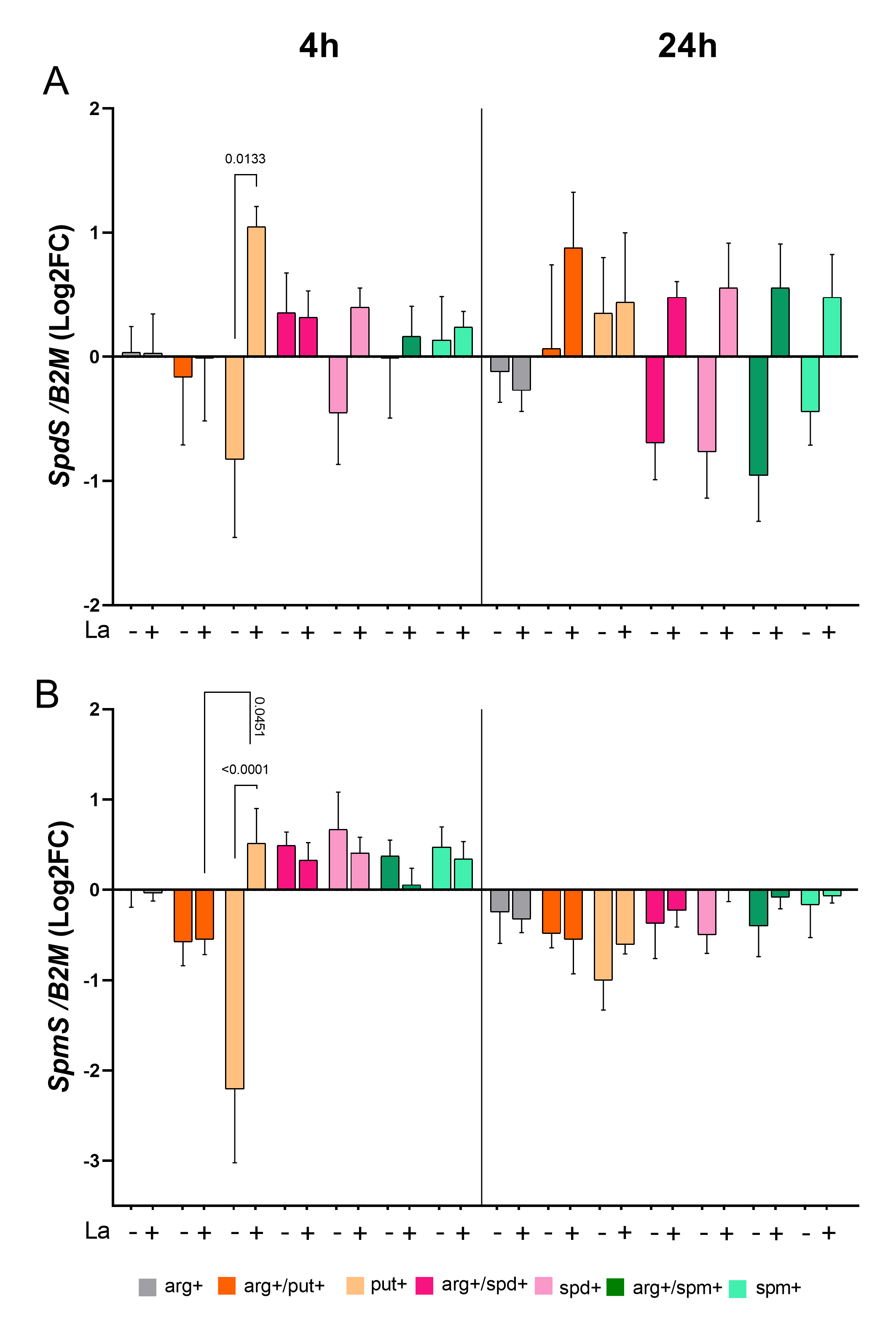

Supplement: S4 Fig — Macrophages were supplemented with putrescine (put+), spermidine (spd+), spermine (spm+) with or without L-arginine (arg+) concomitant to L. amazonensis infection (MOI 5:1) for 4h, and after 24h in complete medium. The RNA was extracted for cDNA conversion and relative quantification of genes SpdS (A) and SpmS (B) by RT-qPCR. Data were normalized using the β-2-microglobulin gene, and the uninfected macrophage arg+ at 4 h was used as a reference in ΔΔCT relative quantification. The bars represent the averages and S.E.M. We performed three independent experiments. Statistical analysis using One-Way ANOVA with mixed-effects, post-hoc test Sidak’s multiple comparisons. (TIF) [file pone.0283696.s004.tif]

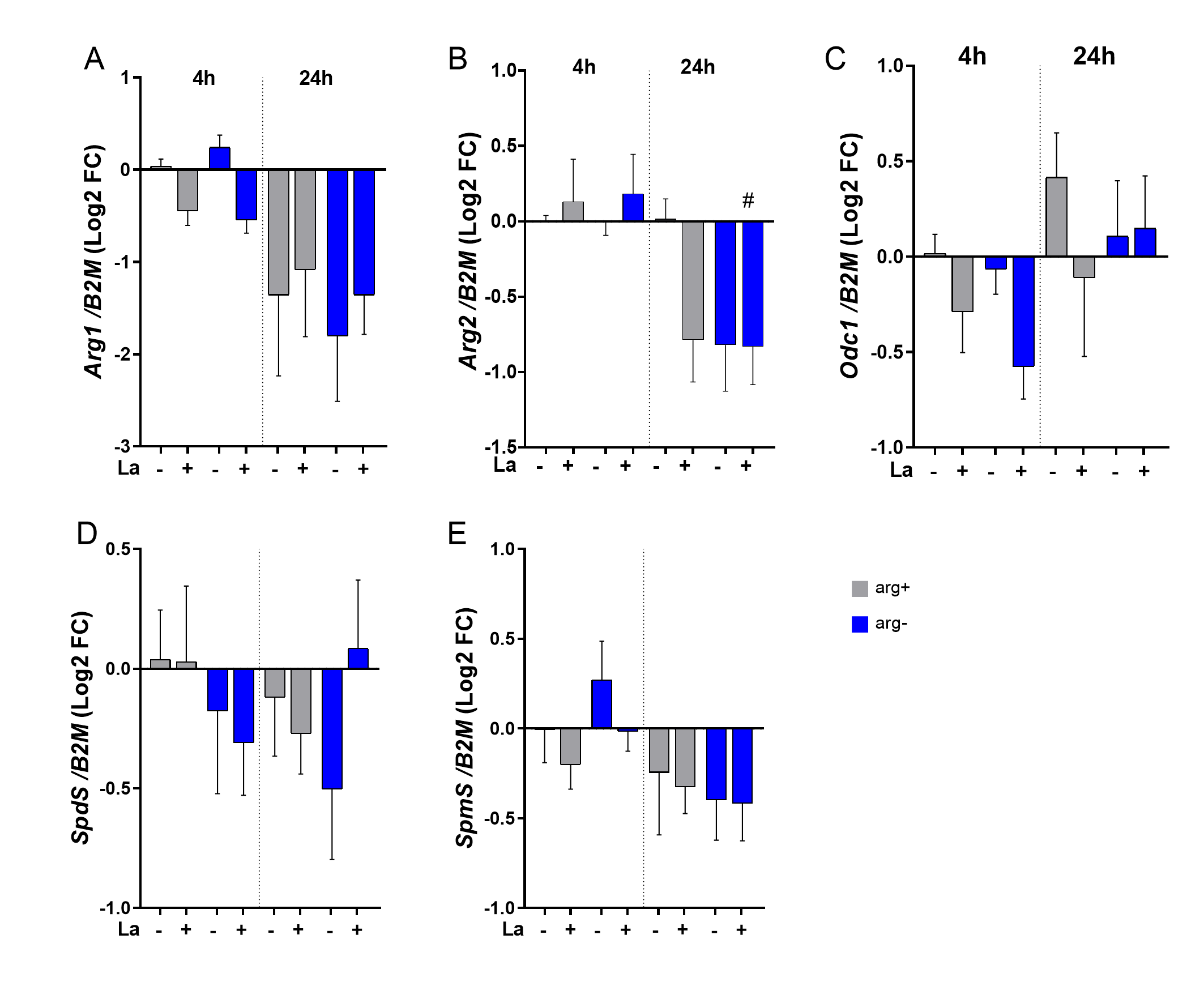

Supplement: S5 Fig — Macrophages were deprived of L-arginine (arg-) or supplemented with L-arginine (arg+) concomitant to L. amazonensis infection (MOI 5:1) for 4h and 24h. Relative quantification of Arg1 (A), Arg2 (B), Odc1 (C), SpdS (D), and SpmS (E) transcripts was performed by RT-qPCR. Data were normalized using the β-2-microglobulin gene, and uninfected macrophage arg+ at 4 h was used as a reference for ΔΔCT relative quantification. The bars represent the averages and S.E.M. We performed three independent experiments. Statistical analysis using One-Way ANOVA with mixed-effects, post hoc test Sidak’s multiple comparisons. #: p≤0.05 for the comparison between 4h vs. 24h. (TIF) [file pone.0283696.s005.tif]

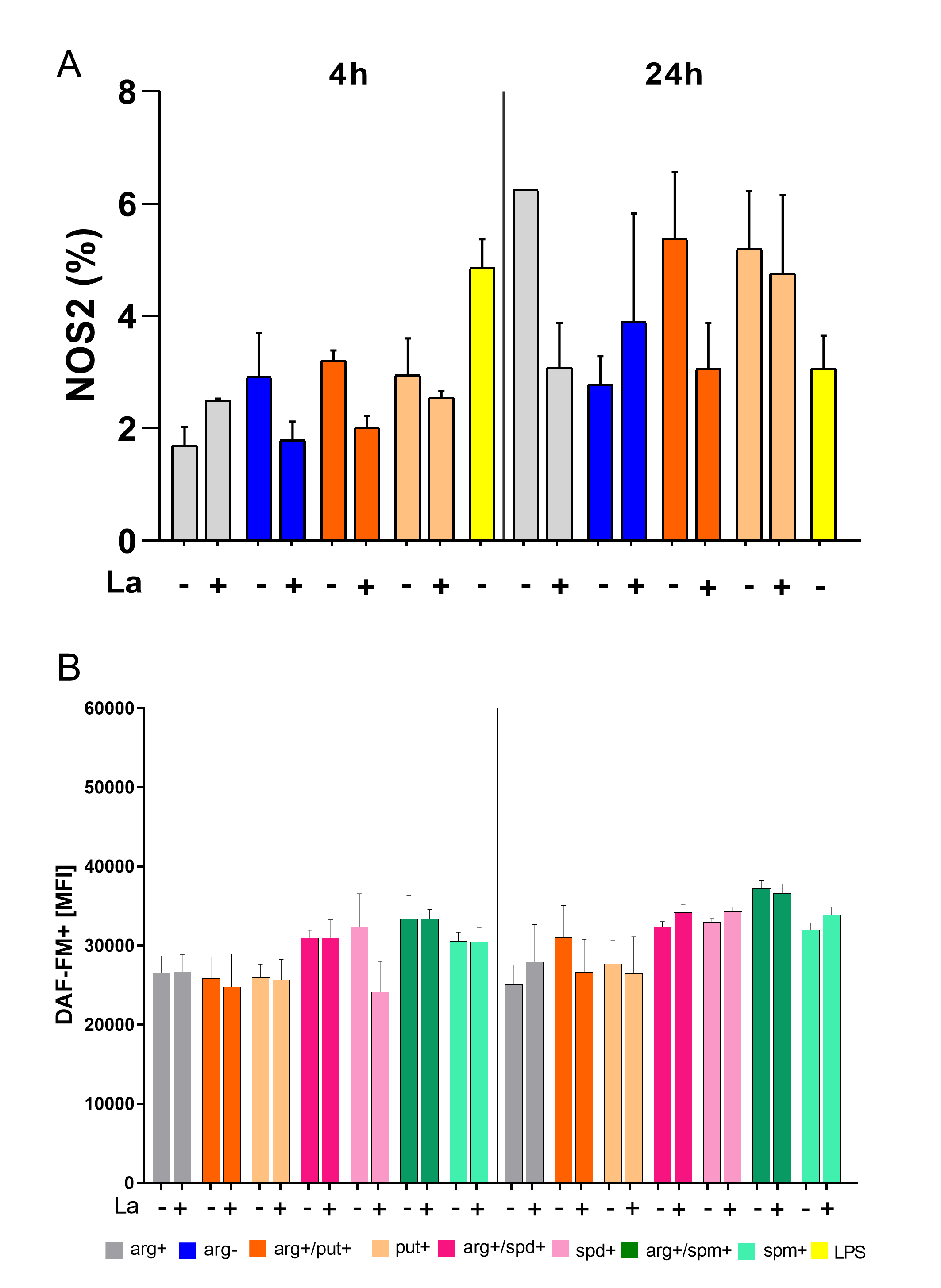

Supplement: S6 Fig — Macrophages (3x10^6^) (A) and (1x10^6^) (A-B) were supplemented with putrescine (put+), spermidine (spd+), spermine (spm+) with or without L-arginine (arg+) concomitant to L. amazonensis infection (MOI 5:1) or stimulated with LPS for 4h, and after 24h in complete medium. The samples were stained with anti-NOS2 for flow cytometry analysis of intracellular levels of NOS2 (A) or DAF-FM for flow cytometry analysis of DAF-FM MFI (B). The bars represent the averages and S.E.M. We performed three independent experiments. Statistical analysis using One-Way ANOVA with mixed-effects, post-hoc test Sidak’s multiple comparisons. (TIF) [file pone.0283696.s006.tif]

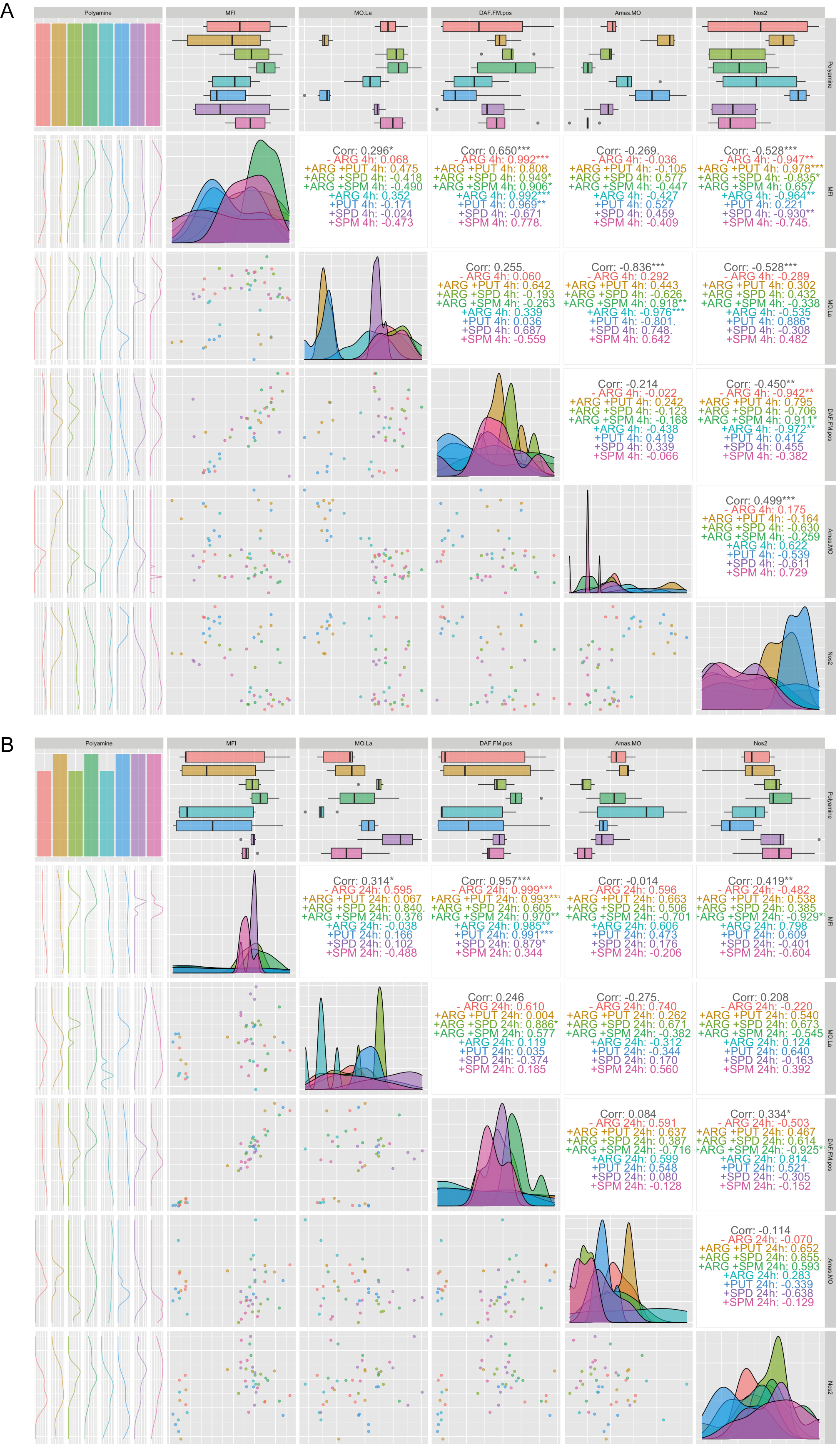

Supplement: S7 Fig — The correlation analysis using ggpairs plot matrix of the percentage of infected macrophage (MO.La), amastigote per infected macrophage (Amas.MO), percentage of NO producing cells (DAF-FM.pos), Mean of fluorescence intensity of NO production (MFI) and Nos2 expression. The correlation plots were generated by GGally (1.5.0) or ggstatplot (0.9.0), the proper correlation tests were conducted with an established p of 0.05. (TIF) [file pone.0283696.s007.tif]

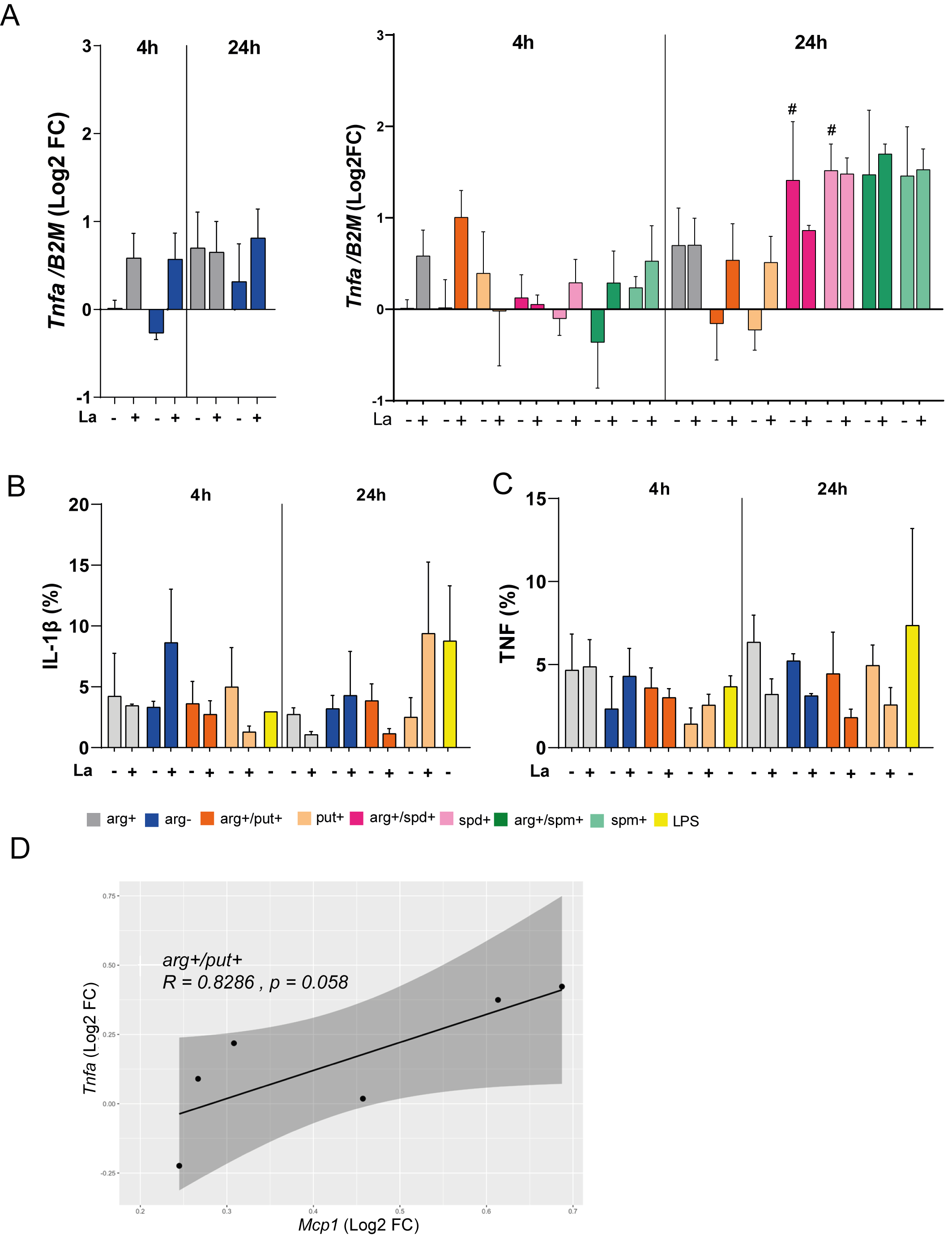

Supplement: S8 Fig — Macrophages were supplemented with putrescine (put+), spermidine (spd+), spermine (spm+) with or without L-arginine (arg+) concomitant or not to L. amazonensis infection (MOI 5:1) or stimulated with LPS for 4h, and after 24h in complete medium. RNA was extracted for cDNA conversion and relative quantification of genes Tnf (A) by RT-qPCR. Data were normalized using the β-2-microglobulin gene, and uninfected macrophage arg+ at 4h was used as a reference for DDCT relative quantification. The samples were stained with APC-anti-IL1B (B) or PE-anti-TNF (C) for flow cytometry analysis of intracellular levels of IL1B or TNF. (TIF) [file pone.0283696.s008.tif]
